# Vitamin D intake as well as circulating 25-hydroxyvitamin D level and risk for the incidence and recurrence of colorectal cancer precursors: A meta-analysis

**DOI:** 10.3389/fmed.2022.877275

**Published:** 2022-08-25

**Authors:** Li-liangzi Guo, Si-si Chen, Li-xian Zhong, Kai-yin He, Yu-ting Li, Wei-wei Chen, Qiu-ting Zeng, Shao-hui Tang

**Affiliations:** Department of Gastroenterology, The First Affiliated Hospital, Jinan University, Guangzhou, China

**Keywords:** Vitamin D, 25-hydroxyvitamin D, colorectal adenoma, sessile serrated adenoma/polyp, meta-analysis

## Abstract

**Objective:**

Vitamin D consumption and circulating 25(OH)D level are associated with decreased risk of colorectal cancer (CRC) and colorectal adenoma (CRA), but few studies have assessed their relationship with the incidence and recurrence of CRC precursors. Therefore, we performed this meta-analysis to further evaluate the association.

**Methods:**

We searched PubMed, Web of Science, Scopus and Embase databases in English until August 2021. Studies evaluating the association of vitamin D intake and circulating 25(OH)D level with risk of CRC precursors were included. A random-effects model was used to pool the risk estimates.

**Results:**

A total of 48 studies were selected for inclusion. The CRC precursors incidence was negatively correlated with total vitamin D intake (RR = 0.84 95%CI: 0.80–0.88) and circulating 25(OH)D level (RR = 0.79 95%CI: 0.67–0.92). However, vitamin D intake and circulating 25(OH)D level did not show significant effects on the risk of CRC precursors recurrence. For dose-response analysis, evidence of a linear association was found between CRC precursors incidence and circulating 25(OH)D level, and the risk decreased by 14% per 10 ng/ml increment of circulating 25(OH)D level (RR = 0.86 95% CI: 0.75–0.99).

**Conclusion:**

Vitamin D intake and circulating 25(OH)D level can play an effective role in reducing the risk of incidence of CRC precursors. However, they have not prevented the recurrence of CRC precursors.

## Introduction

Colorectal cancer is the third leading cancer in the world, with over 1.8 million new cases and 915,880 deaths worldwide (1). The cause is multifactorial including interaction of age, environmental, and genetic factors (2). Different lifestyles have also been shown to have a clear association with CRC. Obesity, sedentary behavior, smoking, alcohol, red and processed meat are associated with increased risk of CRC, while regular physical activity, fish, fruit and vegetables, fiber, folate, calcium, dairy products and vitamin are associated with decreased risk of CRC (3). This shows the important role of nutrition plays as a causal or protective role in the development of CRC. Therefore, screening for validated nutrients is an important adjuvant strategy in CRC prevention.

Vitamin D is one of the essential nutrients for the human body. With its deficiency, it has been linked to skeletal diseases such as fracture (4), bone metabolism (5), and osteoporosis (6) in the past. Because it affects a broad range of health outcomes, vitamin D has been extensively studied of its importance, vitamin D has been extensively studied. Vitamin D is mainly produced through sun exposure and acquired through dietary products and supplements. As the metabolite of vitamin D in the liver, 25(OH)D is widely used to assessed the status of vitamin D (7). A recent umbrella review of 107 systematic reviews and 74 meta-analyses suggested that higher circulating vitamin D level was associated with decreased risk of cancer (breast/colorectal/rectal/sporadic), cardiovascular disease, hypertension, ischemic disease, stroke, Alzheimer’s disease, depression, tuberculosis, metabolic syndrome, diabetes, fractures, CKD mortality and all-cause mortality (8).

The role of vitamin D in the prevention of CRC was first proposed in 1980 (9). *In vitro* experiments have demonstrated the anti-tumor effect of vitamin D, especially in CRC (10). Since then, extensive observational (11, 12), RCT (13), and meta-analysis (14–16) studies have been conducted to evaluate the association of vitamin D intake and/or circulating 25(OH)D level in reducing the risk of CRC incidence, recurrence, survival and mortality. A previous study has shown that vitamin D intake plays a protective role in the development of CRC and a 10ng increment in circulating 25(OH)D level may decreased the risk of CRC by 26% (17).

The development of CRC progresses through three pathways, adenoma-carcinoma sequence, serrated pathway and inflammatory pathway (18). Several acquired genetic and epigenetic changes of normal glandular epithelial cells transformed them into invasive colorectal carcinoma, such as chromosomal instabilities (CIN) (19), microsatellite instabilities (MSI) (20), DNA methylation (21), and APC mutation (22). It is estimated that 85–90% of the sporadic CRC progressed from the adenomatous polyp. Another 10–15% from serrated polyp (23). However, hyperplastic polyp, the most prevalent type of serrated polyp, rarely become cancerous (24). Although most of the early stage CRC and CRC precursors can be detected and removed under colonoscopy, nearly half of the patients recurrent within 1 year follow-up (25). So, it is imperative to reduce the incidence and recurrence of CRC precursors to avoid their progression into CRC. Few studies have tried to evaluate the association of vitamin D intake and/or circulating 25(OH)D levels in reducing the risk of CRC precursors. Epidemiologic data suggest that vitamin D intake was associated with decreased risk of colorectal neoplasia. A previous meta-analysis showed that both vitamin D intake and circulating 25(OH)D levels were inversely correlated with colorectal incidence and recurrent adenomas (26). However, recent RCTs have shown that supplementary vitamin D did not significantly reduce the risk of recurrent CRC precursors (27, 28). Some of the present meta-analyses aimed to elucidate the association between vitamin D intake and colorectal adenoma incidence, they did not mention the history of adenoma so they reported the relative risk of incidence and recurrence together (29). In addition, some only assessed the common effects of vitamin D with calcium (25).

To better understand this relationship, we combined all published studies on the association between vitamin D intake/circulating 25(OH)D level and risk of incidence and recurrence of CRC precursors to perform this systematic review and dose-response meta-analysis.

## Materials and methods

### Design

The protocol of this study was registered in PROSPERO (CRD42021273038). This systematic review and meta-analysis were reported according to PRISMA updated guidelines and PRISMA 2020 27-item checklist (30). We employed the PICO format to answer the research question: “Are vitamin D intake and circulating 25-hydroxyvitamin D level correlated with the risk of the incidence and recurrence of CRC precursors.” Population: Adults with CRA or serrated precursors of CRC (TSA and SSA/P), first-diagnosed or recurrent; adults with only hyperplastic polyps were not included in this study. Intervention: Vitamin D intake (total, dietary and supplementary) or serum/plasma 25(OH)D level. Comparison: Adults without CRA or serrated lesion in the first inspection or follow-up colonoscopy. Outcome: The incidence and recurrence of CRC precursors.

### Search strategy

We searched PubMed, Web of Science, Scopus and Embase database in English up to August 2021. The following search terms were used: “vitamin D” or “25(OH)D” or “25-hydroxyvitamin D”; AND: “colorectal adenoma” or “colorectal polyp” or “colorectal lesion” or “colorectal neoplasm” or “colorectal tumor” or “colorectal carcinoma” or “colorectal cancer.” Titles and abstracts were screened independently by two reviewers (Li-liangzi Guo and Si-si Chen) to exclude irrelevant articles. Duplicated articles were excluded and then full texts were retrieved for intensive reading if it met the inclusion criteria, in order to increase the potentially relevant articles.

### Study selection

Inclusion criteria: (1) adults >18 years of age; (2) study design of randomized controlled trial (RCT) or observational (cohort studies, case-control studies, or cross-sectional studies) that investigated the association between vitamin D intake, circulating 25(OH)D level and risk of incidence or recurrence of CRC precursors; (3) diagnosis: CRC precursors that were determined by histology (adenomatous polyp, traditional serrated adenoma and sessile serrated adenoma/polyp); (4) studies that reported the risk estimates (RR, OR, or HR) with 95% CIs or calculable original data.

Exclusion criteria: (1) animal and *in vitro* studies;(2) non-English papers; (3) studies of children, adolescents or pregnant women; (4) including adults with the previous history of adenoma to analyze the incidence of CRC precursors; (5) combined analysis with CRC or hyperplastic polyp; (6) non-original papers (reviews, editorials or commentaries); (7) meta-analysis studies; (8) studies that did not provide enough data on vitamin D intake/circulating 25(OH)D level and risk estimates; (9) duplicate reports and abstracts; (10) studies that investigated the risk, metastasis, survival and prognosis of CRC.

### Data extraction

Two investigators (Li-xian Zhong and Kai-yin He) independently extracted the data. Any disagreements between the 2 investigators were resolved through consulting the third investigator (Shao-hui Tang). The following information of included studies was extracted: name of the first author, publication year, country, study design, population characteristics (sample size, age and sex), source of vitamin D intake (total/dietary/supplementary), circulating 25-hydroxyvitamin D level, incidence or recurrence of CRC precursors, type of colorectal lesion (high risk or low risk), the corresponding risk estimates with 95% CIs. High-risk adenomas include large size (≥10 mm), high-grade dysplasia, villous component, or multiple adenomas (≥3). For studies that reported both crude and adjusted estimates, we used the adjusted estimates of the greatest degree for this meta-analysis. For dose-response analysis, the distribution of cases and controls, person-years or non-cases, and risk estimates with 95% CIs for >3 categories. The median level of each category was assigned to the corresponding RR for the study. For open-ended categories, we assumed the length of the interval to be the same as that of the closest interval.

### Quality assessment

Two investigators (Yu-ting Li and Wei-wei Chen) independently assessed the study quality. We adopted two different tools to assess the quality of included studies. For observational studies, the Risk of Bias in Non-randomized Studies of Interventions (ROBINS-I) tool was used and studies were evaluated in seven domains: bias due to confounding, bias in the selection of participants, bias in the measurement of interventions, bias due to departures from intended interventions, bias due to missing data, bias in the measurement of outcomes, bias in the selection of the reported result. Studies with low risk of bias in all seven domains were considered as low risk; studies with low or moderate risk in all domains were considered as moderate risk of bias; studies with serious risk or critical risk in at least one domain were considered as serious risk of bias or critical risk of bias respectively (31).

For RCT studies, the risk of bias was assessed by Cochrane Collaboration’s tool and then exported by Review Manager software (RevMan version 5.3). Studies were judged as high risk, low risk, or unclear risk in the six following domains: sequence generation, allocation concealment, blinding of participants, personnel and outcome assessors, incomplete outcome data, selective outcome reporting and other sources of bias (32).

### Statistical analysis

The results were expressed in terms of RR and 95% CI for the highest vs. lowest category of vitamin D intake or circulating 25(OH)D level. The natural log-transformed OR/RR/HRs of each study were pooled using the inverse variance method (DerSimonian and Laird) (33) with a random-effects model (34). Studies comparing for the lowest vs. highest category were re-calculated using Jan Hamling et al. methodology (35). To assess the heterogeneity of individual studies, Cochran’s *Q*-test was used and quantified by I^2^ statistics. I^2^ > 50% and *P* < 0.1 were considered large heterogeneity (36). Sensitivity analysis was conducted to evaluate the stability of the results. Each time one study was omitted to evaluate the risk estimate. Subgroup analyses were further performed according to study design, patient sex, lesion type, and lesion location to explore the potential source of heterogeneity, if data were permitted. Begg’s test, as well as funnel plots, were used to assess publication bias if ≥ 10 studies are available.

If more than 2 observational studies are available, dose-response analysis was conducted to estimate the trend of different categories of vitamin D intake or circulating 25(OH)D level and risk of CRC precursors using generalized least squares (GLST) modeling (37). Both linear and non-linear dose-response analyses were performed. We used restricted cubic spline models with 3 knots at the 10th, 50th, and 90th percentiles of the distribution to construct the non-linear dose-response curve (38).

All statistical analysis was conducted using the STATA software package (version 12.0, Stata Corp., College Station, TX, United States), and the significance difference was defined as *P* < 0.05 by a two-tailed test.

## Results

### Search results and study characteristics

**FIGURE 1:**
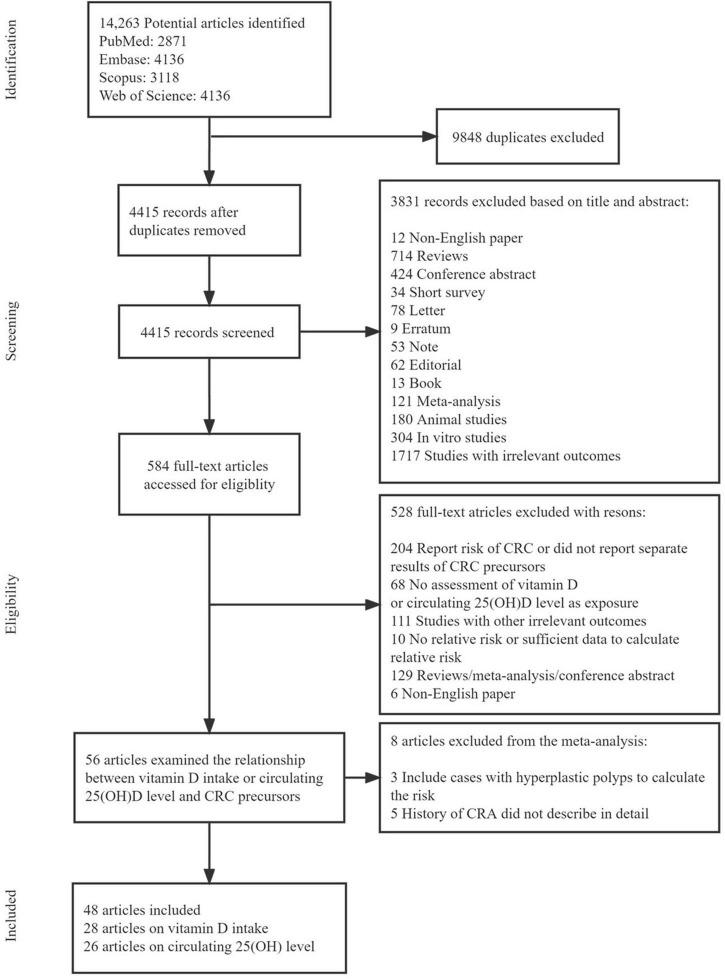
Flow chart of the studies included in this meta-analysis.

Figure 1 shows the flow diagram of the detailed selection process of the included studies. A total of 14,263 potentially relevant articles were initially retrieved. After excluding 9848 duplicate articles, 4415 articles were screened for title and abstract and then 584 articles remained for full-text review. Among them, 528 articles were excluded (204 only report the risk of CRC or did not report separate results of CRC precursors, 62 had no assessment of vitamin D or circulating 25(OH)D as exposure, 111 studied other irrelevant outcomes, 20 had no relative risk or sufficient data to calculate relative risk, 129 were reviews/meta-analysis/conference abstract, and 6 were non-English paper). Therefore, 56 articles were further assessed for inclusion and 8 articles were excluded (3 included cases with hyperplastic polyps to calculate the risk and 5 did not describe detailed information of CRA history). Finally, 48 eligible articles were included in this systematic review and meta-analysis: 28 articles evaluated the association of vitamin D intake on the risk of CRC precursors occurrence (incidence/recurrence) (27, 28, 39–64), 26 articles evaluated the association between circulating 25(OH)D level and risk of CRC precursors occurrence (incidence/recurrence) (28, 39, 43, 47, 50, 57, 65–84). Supplementary Table 4 summarized the main characteristics of the included studies.

### Quality assessment

The quality assessments of the included studies were shown in Supplementary Tables 1, 2. According to the ROBINS-I tool, all 36 observational studies were assessed to have a moderate risk of bias. For 12 RCT studies, all articles were ranked as low risk of bias in randomization, allocation concealment, and selective reporting (Supplementary Figure 1).

### Highest vs. lowest category meta-analysis

#### Vitamin D intake and the risk of colorectal cancer precursors incidence

**FIGURE 2:**
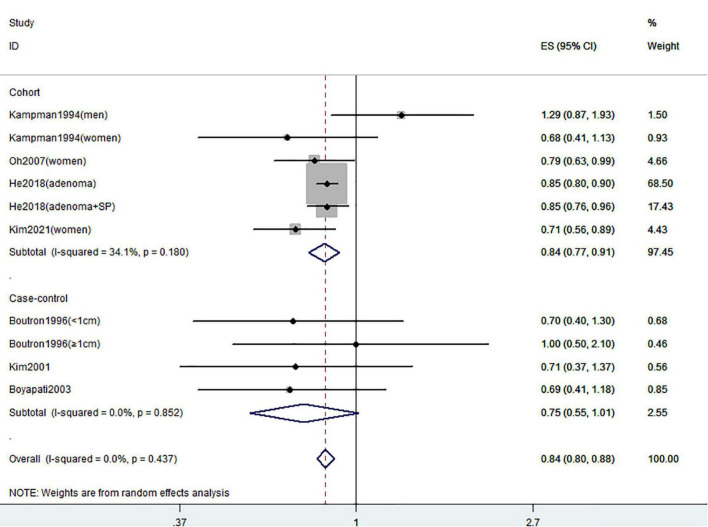
Random-effects meta-analysis of studies that examined total vitamin D intake and risk of CRC precursors incidence. ES, effect size.

**FIGURE 3:**
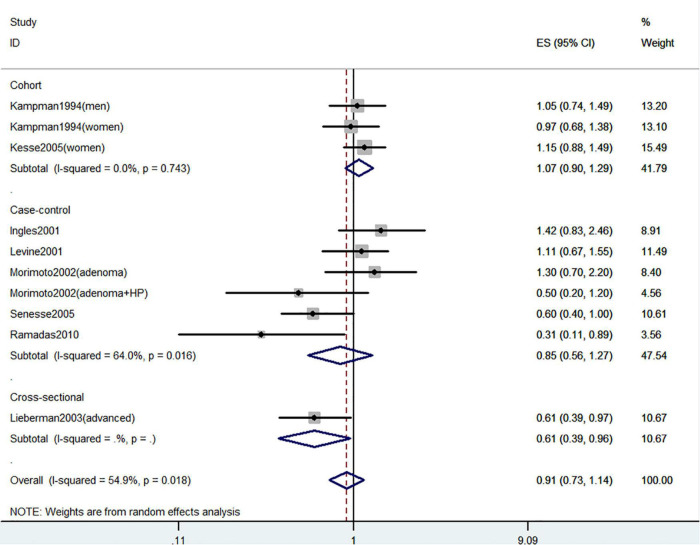
Random-effects meta-analysis of studies that examined dietary vitamin D intake and risk of CRC precursors incidence. ES, effect size.

**FIGURE 4:**
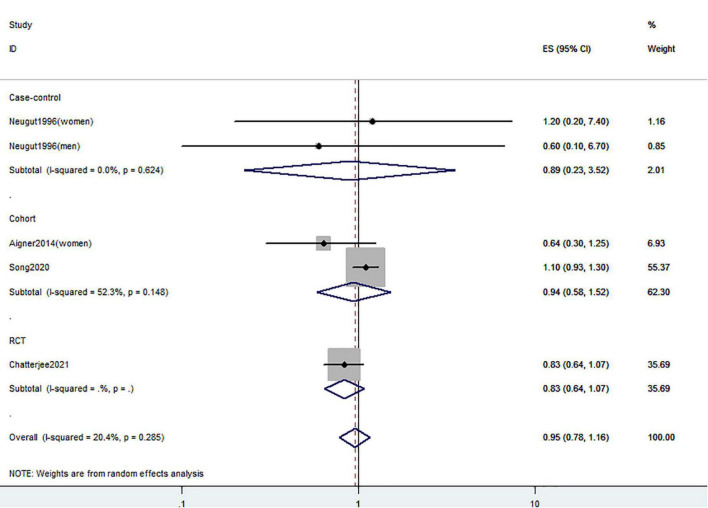
Random-effects meta-analysis of studies that examined supplementary vitamin D intake and risk of CRC precursors incidence. ES, effect size.

Figures 2–4 showed the risk of CRC precursors incidence and total vitamin D intake (*n* = 10), dietary vitamin D intake (*n* = 10), and supplementary vitamin D intake (*n* = 5), respectively. The pooled summary effect size showed that total vitamin D intake reduced the risk of CRC precursors incidence by 16% (RR = 0.84 95% CI: 0.80–0.88, *P* < 0.001, I^2^ = 0%). However, no significant association between dietary vitamin D (RR = 0.91 95% CI: 0.73–1.14, *P* = 0.41, I^2^ = 54.9%) and supplementary vitamin D (RR = 0.95 95% CI: 0.78–1.16, *P* = 0.64, I^2^ = 20.4%) intake with CRC precursors was observed. The results of subgroup analysis were summarized in Table 1 and shown in Supplementary Figure 2. In the case of incidence of CRC precursors, subgroup analysis showed that total vitamin D intake has similar effects in low-risk (RR = 0.88 95% CI: 0.80–0.96, *P* = 0.006, I^2^ = 11%) and high-risk lesion (RR = 0.75 95% CI: 0.69–0.81, *P* < 0.001, I^2^ = 0%). When stratified by sex, an inverse association was observed for intake of total vitamin D in women (RR = 0.74 95% CI: 0.64–0.87, *P* < 0.001, I^2^ = 0%) but not in men (RR = 1.29 95% CI: 0.87–1.92, *P* = 0.21). When stratified by lesion location, total vitamin D intake reduced the risk of incidence of CRC precursors in distal (RR = 0.74 95% CI: 0.65–0.85, *P* < 0.001, I^2^ = 29.9%) and proximal colon lesion (RR = 0.90 95% CI: 0.84–0.97, *P* = 0.008, I^2^ = 0%) more significantly than rectum lesion (RR = 0.84 95% CI: 0.64–1.09, *P* = 0.18, I^2^ = 45.6%). When stratified by study design, an inverse association was observed in cohort study (RR = 0.84 95% CI: 0.77–0.91, *P* < 0.001, I^2^ = 34.1%) rather than in case-control study (RR = 0.75 95% CI: 0.55–1.01, *P* = 0.06, I^2^ = 0%). Furthermore, CRC incidence was inversely associated with total vitamin D intake in American populations (RR = 0.83 95% CI: 0.78–0.90, *P* < 0.001, I^2^ = 16.7%) but not in European populations (RR = 0.81 95% CI: 0.51–1.27, *P* = 0.36, I^2^ = 0%).

**TABLE 1:** Summary of results.

Occurrence	Exposure	Subgroups		No. of studies	Pooled RR (95% CI)	*P*-value	Heterogeneity		Begg’s test
							I^2^ (%)	*P*_*h*_	
Incidence	Total vitamin D intake	All studies		10	0.84 (0.80–0.88)	**<0.001**	0	0.44	0.64
		Design	Cohort	6	0.84 (0.77–0.91)	**<0.001**	34.1	0.18	
			Case-control	4	0.75 (0.55–1.01)	0.06	0	0.85	
		Geographic location	United States	8	0.83 (0.78–0.90)	**<0.001**	16.7	0.30	
			Europe	2	0.81 (0.51–1.27)	0.36	0	0.45	
		Sex	Men	1	1.29 (0.87–1.92)	0.21	NA	NA	
			Women	3	0.74 (0.64–0.87)	**<0.001**	0	0.76	
		Lesion type	Low-risk	4	0.88 (0.80–0.96)	**0.006**	11.0	0.34	
			High-risk	4	0.75 (0.69–0.81)	**<0.001**	0	0.57	
		Lesion location	Distal colon	3	0.74 (0.65–0.85)	**<0.001**	29.9	0.24	
			Proximal colon	2	0.90 (0.84–0.97)	**0.008**	0	0.80	
			Rectum	3	0.84 (0.64–1.09)	0.18	45.6	0.16	
Incidence	Dietary vitamin D intake	All studies		10	0.91 (0.73–1.14)	0.41	**54.9**	0.02	0.06
		Design	Cohort	3	1.07 (0.90–1.29)	0.44	0	0.74	
			Case-control	6	0.85 (0.56–1.27)	0.42	**64.0**	0.02	
			Cross-sectional	1	0.61 (0.39–0.96)	**0.03**	NA	NA	
		Geographic location	United States	7	0.98 (0.78–1.23)	0.86	37.1	0.15	
			Europe	2	0.85 (0.45–1.61)	0.63	**82.8**	0.02	
			Others	1	0.31 (0.11–0.88)	**0.03**	NA	NA	
		Sex	Men	1	1.05 (0.74–1.49)	0.79	NA	NA	
			Women	2	1.08 (0.88–1.34)	0.46	0	0.45	
		Lesion type	High-risk	2	0.78 (0.48–1.27)	0.32	**56.4**	0.13	
		Lesion location	Colon	1	0.60 (0.40–1.00)	0.03	NA	NA	
			Rectum	1	0.70 (0.30–1.40)	0.36	NA	NA	
Incidence	Supplementary vitamin D intake	All studies		5	0.95 (0.78–1.16)	0.64	20.4	0.29	
		Design	RCT	1	0.83 (0.64–1.07)	0.16	NA	NA	
			Cohort	2	0.94 (0.58–1.52)	0.81	**52.3**	0.15	
			Case-control	2	0.89 (0.23–3.52)	0.87	0	0.62	
		Geographic location	United States	4	0.99 (0.83–1.18)	0.92	14.5	0.32	
			Others	1	0.64 (0.31–1.31)	0.22	NA	NA	
		Sex	Men	1	0.60 (0.10–6.70)	0.63	NA	NA	
			Women	2	0.70 (0.36–1.35)	0.29	0	0.53	
Recurrence	Total vitamin D intake	All studies		5	0.93 (0.78–1.11)	0.44	0	0.53	
		Design	RCT	2	0.90 (0.73–1.11)	0.32	0	0.53	
			Case-control	2	1.08 (0.33–3.50)	0.90	1.7	0.31	
			Cross-sectional	1	1.02 (0.71–1.47)	0.92	NA	NA	
		Geographic location	United States	3	0.93 (0.78–1.11)	0.42	0	0.69	
			Others	2	1.08 (0.33–3.50)	0.90	1.7	0.31	
		Lesion type	High-risk	1	1.34 (0.76–2.39)	0.32	NA	NA	
		Lesion location	Proximal colon	1	0.86 (0.61–1.21)	0.38	NA	NA	
			Distal colon	1	0.82 (0.56–1.19)	0.30	NA	NA	
Recurrence	Dietary vitamin D intake	All studies		6	0.82 (0.64–1.04)	0.10	43.3	0.12	
		Design	RCT	3	0.91 (0.76–1.09)	0.32	0	0.79	
			Case-control	2	0.25 (0.10–0.62)	**0.003**	0	0.47	
			Cross-sectional	1	0.78 (0.54–1.13)	0.18	NA	NA	
		Geographic location	United States	4	0.25 (0.10–0.62)	0.14	0	0.47	
			Others	2	0.88 (0.75–1.04)	**0.003**	0	0.80	
		Sex	Men	1	0.95 (0.64–1.40)	0.80	NA	NA	
			Women	1	0.71 (0.38–1.32)	0.28	NA	NA	
		Lesion type	High-risk	1	1.56 (0.84–2.93)	0.16	NA	NA	
		Lesion location	Proximal colon	1	1.09 (0.78–1.52)	0.61	NA	NA	
			Distal colon	1	0.89 (0.61–1.29)	0.54	NA	NA	
Recurrence	Supplementary vitamin D intake	All studies		10	0.99 (0.92–1.06)	0.80	0	0.57	0.32
		Design	RCT	4	0.99 (0.88–1.10)	0.83	43.6	0.15	
			Cohort	1	0.75 (0.35–1.59)	0.45	NA	NA	
			Case-control	4	0.90 (0.57–1.43)	0.65	0	0.66	
			Cross-sectional	1	1.05 (0.56–1.98)	0.88	NA	NA	
		Geographic location	United States	8	0.97 (0.88–1.06)	0.45	0	0.50	
			Europe	1	1.04 (0.93–1.17)	0.50	NA	NA	
			Others	1	0.83 (0.45–1.52)	0.54	NA	NA	
		Sex	Men	1	1.70 (0.10–22.70)	0.70	NA	NA	
			Women	1	4.10 (0.30–54.90)	0.29	NA	NA	
		Lesion type	High-risk	4	1.11 (0.87–1.42)	0.40	27.2	0.25	
		Lesion location	Proximal colon	1	0.78 (0.63–0.96)	**0.02**	NA	NA	
			Distal colon	1	0.79 (0.62–0.99)	**0.048**	NA	NA	
Incidence	Circulating 25(OH)D level	All studies		19	0.79 (0.67–0.92)	**0.002**	46.9	0.01	0.15
		Design	RCT	1	0.83 (0.64–1.08)	0.16	NA	NA	
			Cohort	3	0.93 (0.58–1.47)	0.74	**59.2**	0.09	
			Case-control	13	0.75 (0.62–0.91)	**0.003**	47.2	0.03	0.07
			Cross-sectional	2	0.84 (0.30–2.35)	0.73	**73.9**	0.05	
		Geographic location	United States	14	0.77 (0.66–0.90)	**0.001**	21.7	0.22	0.38
			Others	5	0.80 (0.54–1.19)	0.27	**75.6**	0.003	
		Sex	Men	6	0.91 (0.68–1.21)	0.52	27.7	0.09	
			Women	6	0.68 (0.42–1.09)	0.11	**68.7**	0.007	
		Lesion type	High-risk	7	0.74 (0.62–0.88)	**0.001**	0	0.53	
			Low-risk	6	0.57 (0.47–0.69)	**<0.001**	0	0.70	
		Lesion location	Proximal colon	5	0.69 (0.46–1.03)	0.07	**71.9**	0.007	
			Distal colon	6	0.77 (0.63–0.95)	**0.02**	23.3	0.26	
			Rectum	3	0.83 (0.51–1.37)	0.47	0	0.47	
Recurrence	Circulating 25(OH)D level	All studies		13	0.95 (0.86–1.04)	0.24	21.2	0.23	**0.009**
		Design	RCT	11	0.93 (0.83–1.04)	0.20	33.1	0.13	**0.008**
			Case-control	1	0.94 (0.54–1.64)	0.83	NA	NA	
			Cross-sectional	1	1.05 (0.75–1.46)	0.77	NA	NA	
		Geographic location	United States	12	0.92 (0.82–1.04)	0.20	24.9	0.20	**0.006**
			Europe	1	0.99 (0.90–1.09)	0.84	NA	NA	
		Sex	Men	6	0.95 (0.80–1.14)	0.58	0	0.80	
			Women	6	0.82 (0.64–1.06)	0.13	0	0.48	
		Lesion type	High-risk	9	1.00 (0.87–1.15)	0.99	0	0.78	
		Lesion location	Proximal colon	2	0.89 (0.58–1.37)	0.59	**75.8**	0.04	
			Distal colon	2	1.05 (0.83–1.32)	0.69	0	0.51	

High risk: large size (≥10 mm), high-grade dysplasia, villous component, or multiple adenomas (≥3); P_h_: P value for heterogeneity; values with statistical significant were presented with bold type.

#### Vitamin D intake and the risk of colorectal cancer precursors recurrence

**FIGURE 5:**
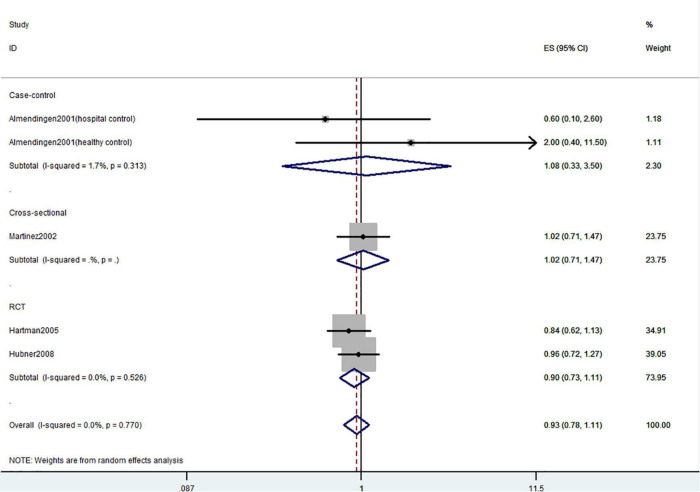
Random-effects meta-analysis of studies that examined total vitamin D intake and risk of CRC precursors recurrence. ES, effect size.

**FIGURE 6:**
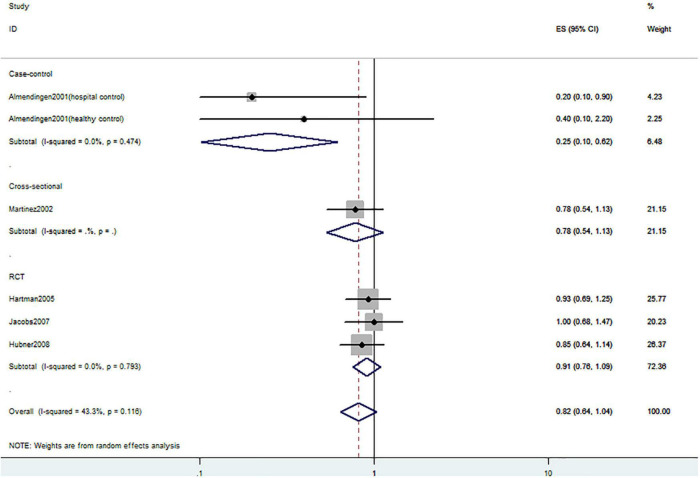
Random-effects meta-analysis of studies that examined dietary vitamin D intake and risk of CRC precursors recurrence. ES, effect size.

**FIGURE 7:**
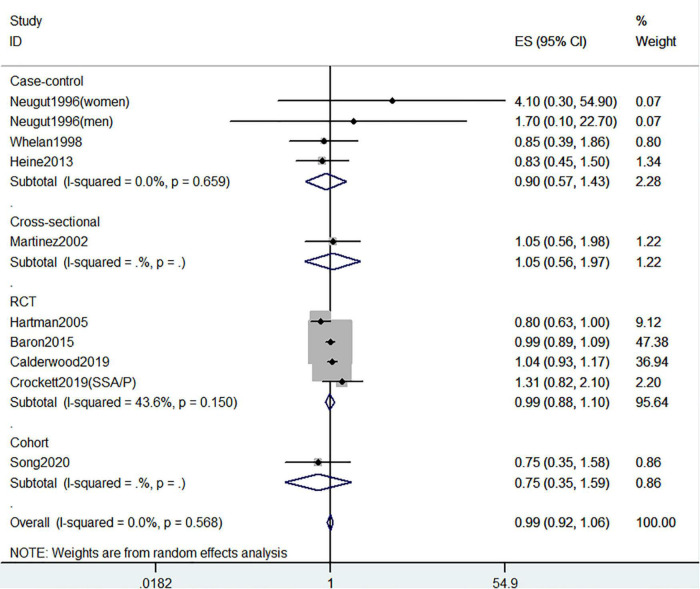
Random-effects meta-analysis of studies that examined supplementary vitamin D intake and risk of CRC precursors recurrence. ES, effect size.

Figures 5–7 showed the risk of CRC precursors recurrence and total vitamin D intake (*n* = 5), dietary vitamin D intake (*n* = 6), and supplementary vitamin D intake (*n* = 9), respectively. The pooled summary effect size showed no significant association between total vitamin D (RR = 0.93 95% CI: 0.78–1.11, *P* = 0.44, I^2^ = 0%), dietary vitamin D (RR = 0.82 95% CI: 0.64–1.04, *P* = 0.10, I^2^ = 43.3%), and supplementary vitamin D (RR = 0.99 95% CI: 0.92–1.06, *P* = 0.80, I^2^ = 0%) intake with the risk of CRC precursors.

#### Circulating 25(OH)D level and the risk of colorectal cancer precursors incidence

**FIGURE 8:**
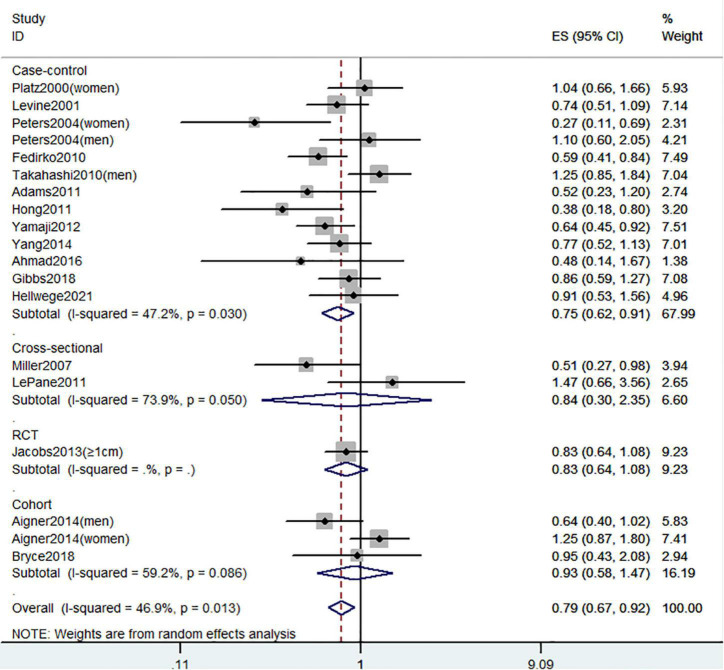
Random-effects meta-analysis of studies that examined circulating 25(OH)D level and risk of CRC precursors incidence. ES, effect size.

Figure 8 showed the risk of CRC precursors incidence and circulating 25(OH)D level (*n* = 19). The results showed that a high level of circulating 25(OH)D decreased 21% risk of CRC precursors (RR = 0.79 95% CI: 0.67–0.92, *P* = 0.002, I^2^ = 46.9%). Subgroup analysis showed that circulating 25(OH)D level has similar effects in low-risk (RR = 0.57 95% CI: 0.47–0.69, *P* < 0.001, I^2^ = 0%) and high-risk lesion (RR = 0.74 95% CI: 0.62–0.88, *P* = 0.001, I^2^ = 0%). When stratified by sex, no significant association was observed in both men (RR = 0.91 95% CI: 0.68–1.21, *P* = 0.52, I^2^ = 27.7%) and women (RR = 0.68 95% CI: 0.42–1.09, *P* = 0.11, I^2^ = 68.7%). When stratified by lesion location, an inverse association was observed for circulating 25(OH)D level in distal colon (RR = 0.77 95% CI: 0.63–0.95, *P* = 0.02, I^2^ = 23.3%) but not in proximal colon (RR = 0.69 95% CI: 0.46–1.03, *P* = 0.07) and rectum (RR = 0.83 95% CI: 0.51–1.37, *P* = 0.47). In addition, the protective effect of high circulating 25(OH)D level for CRC precursors risk was found in case-control study (RR = 0.75 95% CI: 0.62–0.91, *P* = 0.003, I^2^ = 47.2%) and American populations (RR = 0.77 95% CI: 0.66–0.90, *P* = 0.001, I^2^ = 21.7%).

#### Circulating 25(OH)D level and the risk of colorectal cancer precursors recurrence

**FIGURE 9:**
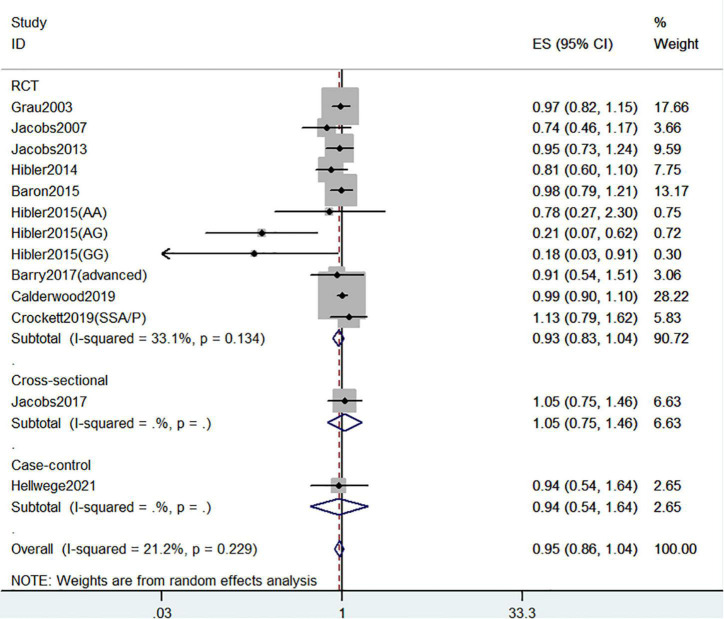
Random-effects meta-analysis of studies that examined circulating 25(OH)D level and risk of CRC precursors recurrence. ES, effect size.

Figure 9 showed the risk of CRC precursors recurrence and circulating 25(OH)D level (*n* = 13). However, there was no significant association between circulating 25(OH)D level and risk of CRC precursors (RR = 0.95 95% CI: 0.86–1.04, *P* = 0.24, I^2^ = 21.2%).

### Dose-response meta-analysis

**FIGURE 10:**
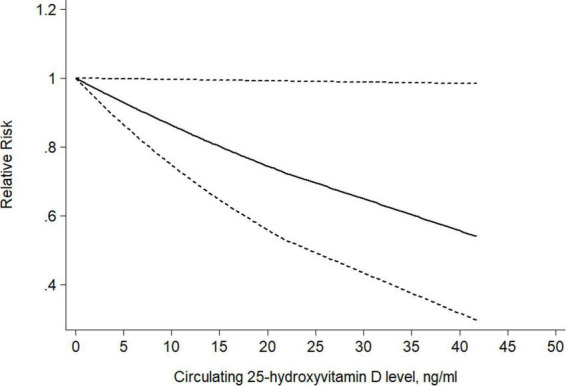
Dose-response relationship between circulating 25(OH)D level and risk of CRC precursors incidence. Weights are from the fixed-effects analysis. Solid line represents the linear trend and short dashes line represent the 95% CI.

Seven studies were available for a dose-response analysis of the association between circulating 25(OH)D level and risk of CRC precursors incidence. Both linear and non-linear dose-response analyses were performed. Trend meta-analysis showed a significant negative dose-response relationship in circulating 25(OH)D (*P_–non–linearity_* = 0.39) level from linearity. As shown in Figure 10, we found that per 10ng/ml increment of circulating 25(OH)D level could decrease the risk of colorectal cancer precursors by 14% using the fixed-effect model with no heterogeneity (RR = 0.86 95% CI: 0.75–0.99, *P* = 0.04; *P_*h*_* = 0.96).

### Publication bias and sensitivity analysis

The results of Begg’s test showed no publication bias for most of the outcomes but a publication bias for circulating 25(OH)D level and CRC precursors recurrence (Begg’s *P* = 0.009). The funnel plots were presented in Supplementary Figure 3. We conducted a sensitivity analysis to evaluate the influence of a single study on the overall risk by omitting one study in each turn (Supplementary Table 3). The effect estimated from the sensitivity analyses showed little change which means that the results were stable.

## Discussion

The study is the latest and most comprehensive study focused on the association between vitamin D and circulating 25(OH)D level with the incidence and recurrence of CRC precursors. Decreased risk of CRC precursors incidence was observed for total vitamin D intake and 25(OH)D level rather than dietary vitamin D and supplementary vitamin D intake. These discrepant findings may be attributed to different doses, metabolism, bioavailability of vitamin D and other unadjusted potential confounding factors. However, neither vitamin D nor 25(OH)D level play an effective role in preventing the recurrence of CRC precursors.

Our systematic review and meta-analysis updated and expanded upon the eight previous meta-analysis. The first meta-analysis conducted by Wei et al. (26) showed that both circulating 25(OH)D and vitamin D intake was inversely associated with colorectal adenoma incidence and recurrence. The inconsistent result of recurrent risk may be attributed to the small number of included studies. They involved only 4 epidemiologic studies to assess the recurrent risk with vitamin D intake whereas we involved 12 studies, including 6 RCTs and 6 observational studies. Yin et al. (85) and Fedirko et al. (69) reached a similar conclusion as our study, but they did not assess the effect of vitamin D intake. The two most recent meta-analyses of Choi et al. (86) and Huang et al. (29) both included hyperplastic polyps to assess the association and neither of them separated patients with a history of adenoma from those newly diagnosed. Gandini et al. (87) and Zhang et al. (88) included too few studies to reach a reliable result.

Vitamin D belongs to a group of steroids known as secosteroids. Vitamin D2 (ergocalciferol) and vitamin D3 (cholecalciferol) are the most common forms of vitamin D in human body. They can be obtained from the diet and synthesized through ultraviolet B radiation (89). Vitamin D is activated to calcitriol (1,25-dihydroxyvitamin D3) by two cytochrome P450-mediated (CYP450) hydroxylation steps. First, vitamin D was catalyzed by CYP2R1 in the liver to yield circulating 25(OH)D (90). Then, 25(OH)D is metabolized by CYP27B1 in the kidney to yield calcitriol. Calcitriol then performs its biological functions by binding and activating the nuclear vitamin D receptor (VDR), down-regulating the gene transcription of CYP27B1 and parathyroid hormone (PTH), and inducing the expression of CYP24A1 (10, 91–93).

Vitamin D may decrease the risk of CRC precursors incidence through several mechanisms. VDR, present in the majority cells of the human body and is expressed abundantly in intestinal epithelial cells. Previous studies have shown that VDR is a biomarker for the anti-proliferative effect of vitamin D on CRC (94). *In vitro* studies have shown that calcitriol may paly pro-differentiation effects through increasing the activity of alkaline phosphatase in colorectal cancer cells and colorectal adenoma cell lines (95). Further, it can induce apoptosis in colorectal adenoma and colorectal cancer by up-regulating the expression of pro-apoptotic proteins (96). Moreover, the gut microbiome is believed to be directly involved in colon carcinogenesis (97). Vitamin D has also been reported to regulate the gut microbiome in animal studies (98).

Our systematic review and meta-analysis had several strengths. First, the heterogeneity between studies was low in most of the analyses. Second, we include both RCT and observational studies through a systematic search. Third, an inverse dose-response relationship was observed between circulating 25(OH)D level and CRC precursors incidence, which may strengthen the reliability of the results. Sensitivity analysis was also carried out to investigate the stability of the results. Last, most of the present meta-analyses did not mention the history of adenoma so they report the relative risk of incidence and recurrence together. We conduct this study to analyze the incidence and recurrence of CRC precursors separately. Besides, some meta-analyses assessed the common effects of vitamin D with calcium, but we only assessed the exact effect of vitamin D.

However, there were some limitations to this study. First of all, due to the small number of included studies, further subgroup analyses of lesion type and dose-response analyses of vitamin D intake were not able to be performed. Thus, it is hard to deduce the optimal dosage of vitamin D intake. Second, unpublished researches were not included which may cause potential publication bias. Articles published in non-English language were not included in this study either. Third, although we have included RCT studies that may avoid recall and selection bias, the rest of the case-control studies may not have avoided it. In addition, not all potential confounders were adjusted for in every study, such as sample size, follow-up duration, and study design etc. Fourth, most RCTs were not well-designed according to the guidelines, which vitamin D dose should base on serum 25(OH)D level and should be large enough to increase 25(OH)D concentration (99, 100). Finally, regarding dietary vitamin D intake, because many food frequency tables do not include data on meat, so most of the studies ignored meat as a source of vitamin (101, 102). Furthermore, a recent RCT study suggested that the benefit of supplementary vitamin D intake in preventing advanced colorectal adenomas may vary according to different VDR (65). However, there is little study aimed at this association that data of genetic variants in VDR or vitamin D binding protein (VDBP) were not considered in this study.

## Conclusion

In conclusion, our study clarified a potentially effective role of total vitamin D intake and circulating 25(OH)D levels in reducing the risk of CRC precursors incidence. Although vitamin D intake and circulating 25(OH)D levels had no positive effect in reducing the recurrence risk of CRC precursors, its function in reducing the incidence of CRC precursors is also important in CRC prevention strategies. More large and precise prospective studies are needed to further verify the association and identify the underlying mechanism.

## Data availability statement

The original contributions presented in this study are included in the article/Supplementary material, further inquiries can be directed to the corresponding author/s.

## Ethics statement

Ethical review and approval was not required for the study on human participants in accordance with the local legislation and institutional requirements. Written informed consent for participation was not required for this study in accordance with the national legislation and the institutional requirements.

## Author contributions

L-LG, S-SC, and L-XZ contributed equally to this work. L-LG and S-SC contributed to the conception and design of the study. L-XZ and K-YH extracted the data. Y-TL and W-WC assessed the methodological quality of included studies. L-XZ, S-SC, and Q-TZ contributed to statistical analysis. L-LG drafted the manuscript. S-HT reviewed and revised the manuscript. All authors have read and approved the final manuscript.
