# Supplementary material for: Vitamin D intake as well as circulating 25-hydroxyvitamin D level and risk for the incidence and recurrence of colorectal cancer precursors: A meta-analysis
Source: Front Med (Lausanne). 2022 Aug 25;9:877275. doi: 10.3389/fmed.2022.877275 (PMC9452754; doi:10.3389/fmed.2022.877275)
Supplement: Supplementary file 1 [file Data_Sheet_1.pdf]

Supplementary Table 1. Bias risk of included observational studies assessed by ROBINS-I.

Study	Bias due to confounding	Bias in selection of participants	Bias in measurement of interventions	Bias due to departures from intended interventions	Bias due to missing data	Bias in measurement of outcomes	Bias in selection of the reported result	Overall bias
Kampman et al, 1994	Moderate risk	Low risk	Low risk	Low risk	Low risk	Low risk	Low risk	Moderate risk
Boutron et al, 1996	Moderate risk	Low risk	Moderate risk	Low risk	Moderate risk	Low risk	Low risk	Moderate risk
Neugut, 1996, USA	Moderate risk	Low risk	Moderate risk	Low risk	Low risk	Low risk	Low risk	Moderate risk
Whelan, 1998, USA	Moderate risk	Low risk	Low risk	Low risk	Low risk	Moderate risk	Low risk	Moderate risk
Platz, 2000, USA	Moderate risk	Moderate risk	Low risk	Low risk	Low risk	Moderate risk	Low risk	Moderate risk
Almendingen, 2001, Norway	Low risk	Low risk	Moderate risk	Low risk	Low risk	Low risk	Low risk	Moderate risk
Ingles, 2001, USA	Moderate risk	Low risk	Low risk	Low risk	Low risk	Low risk	Low risk	Moderate risk
Kim, 2001, USA	Moderate risk	Low risk	Low risk	Low risk	Low risk	Moderate risk	Low risk	Moderate risk
Levine, 2001, USA	Moderate risk	Low risk	Low risk	Low risk	Moderate risk	Low risk	Low risk	Moderate risk
Martinez, 2002, USA	Moderate risk	Low risk	Low risk	Low risk	Moderate risk	Low risk	Low risk	Moderate risk
Morimoto, 2002, USA	Moderate risk	Low risk	Low risk	Low risk	Moderate risk	Low risk	Low risk	Moderate risk

Boyapati, 2003, USA	Moderate risk	Low risk	Moderate risk	Low risk	Low risk	Low risk	Low risk	Moderate risk
Grau, 2003, USA	Moderate risk	Low risk	Low risk	Low risk	Moderate risk	Low risk	Low risk	Moderate risk
Liberman, 2003, USA	Moderate risk	Low risk	Low risk	Low risk	Low risk	Low risk	Low risk	Moderate risk
Peters, 2004, USA	Moderate risk	Low risk	Low risk	Low risk	Low risk	Low risk	Low risk	Moderate risk
Kesse et al, 2005	Moderate risk	Low risk	Moderate risk	Low risk	Moderate risk	Low risk	Low risk	Moderate risk
Senesse, 2005, France	Moderate risk	Low risk	Low risk	Low risk	Low risk	Low risk	Low risk	Moderate risk
Miller, 2006, USA	Moderate risk	Low risk	Low risk	Low risk	Moderate risk	Low risk	Low risk	Moderate risk
Oh, 2007, USA	Moderate risk	Moderate risk	Low risk	Low risk	Low risk	Low risk	Low risk	Moderate risk
Fedirko, 2010, USA	Moderate risk	Low risk	Low risk	Low risk	Low risk	Low risk	Low risk	Moderate risk
Ramadas, 2010, Malaysia	Moderate risk	Low risk	Moderate risk	Low risk	Low risk	Low risk	Low risk	Moderate risk
Takahashi, 2010, Japan	Moderate risk	Moderate risk	Low risk	Low risk	Low risk	Low risk	Low risk	Moderate risk
Adams, 2011, USA	Moderate risk	Low risk	Low risk	Low risk	Low risk	Low risk	Low risk	Moderate risk
LePane, 2011, USA	Moderate risk	Low risk	Low risk	Low risk	Low risk	Low risk	Low risk	Moderate risk
Hong, 2012, South Korea	Moderate risk	Low risk	Low risk	Low risk	Low risk	Low risk	Low risk	Moderate risk

Yamaji, 2012, Japan	Moderate risk	Low risk	Moderate risk	Low risk	Low risk	Low risk	Low risk	Moderate risk
Heine, 2012, Netherland	Low risk	Low risk	Low risk	Low risk	Moderate risk	Low risk	Low risk	Moderate risk
Aigner, 2014, Australia	Moderate risk	Low risk	Low risk	Low risk	Low risk	Low risk	Low risk	Moderate risk
Yang, 2014, USA	Moderate risk	Low risk	Moderate risk	Low risk	Low risk	Low risk	Low risk	Moderate risk
Ahmad, 2016, USA	Moderate risk	Moderate risk	Low risk	Low risk	Low risk	Low risk	Low risk	Moderate risk
Jacobs, 2016, USA	Moderate risk	Low risk	Low risk	Low risk	Low risk	Moderate risk	Low risk	Moderate risk
Bryce, 2018, USA	Moderate risk	Low risk	Moderate risk	Low risk	Low risk	Low risk	Low risk	Moderate risk
Gibbs, 2018, USA	Moderate risk	Low risk	Low risk	Low risk	Low risk	Low risk	Low risk	Moderate risk
He, 2018, USA	Moderate risk	Moderate risk	Moderate risk	Low risk	Low risk	Moderate risk	Low risk	Moderate risk
Hellwege, 2021, USA	Moderate risk	Low risk	Moderate risk	Low risk	Low risk	Low risk	Low risk	Moderate risk
Kim, 2021, USA	Moderate risk	Low risk	Low risk	Low risk	Low risk	Moderate risk	Low risk	Moderate risk

Supplementary Table 2. Bias risk of included RCT assessed by Cochrane Collaboration's tool.

Study	Random sequence generation	Allocation concealment	Blinding of participants and researchers	Blinding of outcome assessment	Incomplete outcome data	Selective reporting	Other bias
Hartman, 2005, USA	Low risk	Low risk	Unclear risk	Low risk	Low risk	Low risk	Unclear risk

Jacobs, 2007, USA	Low risk	Low risk	Low risk	Low risk	Low risk	Low risk	Low risk
Hubner, 2008, USA	Low risk	Low risk	Low risk	Low risk	Low risk	Low risk	Low risk
Jacobs, 2013, USA	Low risk	Low risk	Low risk	Low risk	Unclear risk	Low risk	Unclear risk
Hibler, 2014, USA	Low risk	Low risk	Low risk	Low risk	Unclear risk	Low risk	Unclear risk
Baron, 2015, USA	Low risk	Low risk	Low risk	Low risk	Unclear risk	Low risk	Unclear risk
Hibler, 2015, USA	Low risk	Low risk	Low risk	Low risk	Low risk	Low risk	Unclear risk
Barry, 2017, USA	Low risk	Low risk	Low risk	Low risk	Low risk	Low risk	Unclear risk
Calderwood, 2019, Germany	Low risk	Low risk	Low risk	Unclear risk	Unclear risk	Low risk	Unclear risk
Crockett, 2019, USA	Low risk	Low risk	Low risk	Low risk	Unclear risk	Low risk	Unclear risk
Chatterjee, 2021, USA	Low risk	Low risk	Low risk	Low risk	Unclear risk	Low risk	Unclear risk
Song, 2020, USA	Low risk	Low risk	Low risk	Low risk	Low risk	Low risk	Low risk

Supplementary Table 3. The sensitivity analysis of the relationship between circulating 25(OH)D level and risk of CRC precursors recurrence.

Excluded studies	No. of included studies	Heterogeneity		Random-effect model		
		I^2	P_h	RR	95% CI	P
**All studies**						
No	13	21.2%	0.23	0.95	0.86-1.04	0.24
Grau2003	12	27.7%	0.17	0.93	0.83-1.05	0.24
Jacobs2007	12	21.4%	0.23	0.96	0.87-1.05	0.34
Jacobs2013	12	27.8%	0.17	0.94	0.85-1.05	0.26
Hibler2014	12	21.0%	0.24	0.96	0.87-1.06	0.39
Baron2015	12	27.6%	0.17	0.93	0.84-1.04	0.23
Hibler2015(AA)	12	27.1%	0.18	0.94	0.86-1.04	0.26
Hibler2015(AG)	12	0%	0.74	0.97	0.90-1.04	0.35
Hibler2015(GG)	12	4.5%	0.40	0.96	0.89-1.04	0.30
Jacobs2017	12	26.5%	0.18	0.94	0.84-1.04	0.20
Barry2017(advanced)	12	26.5%	0.18	0.94	0.84-1.04	0.20
Calderwood2019	12	24.9%	0.20	0.92	0.82-1.04	0.20
Crockett2019(SSA/P)	12	23.8%	0.21	0.93	0.85-1.03	0.17
Hellwege2021	12	27.8%	0.17	0.94	0.84-1.04	0.25

P_h: P value for heterogeneity.

Supplementary Table 4. Basic characteristics of eligible studies.

Author, year, location	Study design	Study period	Age and sex	No. of cases	No. of controls	Occurrence	Source of vitamin D intake	Vitamin D biochemical measurement	Contrast	OR/RR/HR (95% CI)	Adjustments
Kampman, 1994, USA	Cohort	HPFS: 1986-1990, NHS: 1980-1988	HPFS: 40-75y, M NHS: 30-55y, F	Adenomatous polyp of the left colon or rectum: 331 men, 350 women	Polyp-free control: 9159 men, 8585 women	Incidence	Total	NA	C5 vs C1	Total	Age, total energy Intake, BMI, alcohol consumption, folate, Intake of saturated fat and fiber, Indications for endoscopy, history of endoscopy prior to the study period, and family history.
									HPFS (male)	RR 1.29(0.87-1.93)	
									NHS (female)	RR 0.68(0.41-1.13)	
							Dietary		C5 vs C1	Dietary	
									HPFS (male)	RR 1.05(0.74-1.49)	
									NHS (female)	RR 0.97(0.68-1.38)	
Boutron, 1996, France	Case-control	1985-1990	30-75y, M+F	154 small adenoma and 208	426 polyp-free	Incidence	Total		C5 vs C1		Age, sex, and caloric intake.
									Small adenoma	OR 0.7(0.4-1.3)	

				large adenomas	control				Large adenoma	OR 1.0(0.5-2.1)	
Neugut, 1996, USA	Case-control	1986-1988	35-84y, M+F	297 newly diagnosed adenomas	505 polyp-free controls	Incidence	Supplementary	NA	User vs nonuser	Incidence	Age, leisure activity years of education. dietary fiber intake, dietary fat intake, and years of cigarette smoking (males only).
									Female	OR 1.2(0.2-7.4)	
									Male	OR 0.6(0.1-6.7)	
				198 recurrent adenomas	347 with previous history of polyp	Recurrence			User vs nonuser	Recurrence	
									Female	OR 4.1(0.3-54.9)	
									Male	OR 1.7(0.1-22.7)	
Whelan, 1998, USA	Case-control	1993-1997	Average 65.5y, M+F	183 with recurrent adenomas	265 without recurrent adenomas	Recurrence	Supplementary	NA	User vs nonuser	OR 0.848(0.388-1.857)	Age, gender, and used adenoma recurrence at index colonoscopy as the outcome.

Platz, 2000, USA	Nested case-control	1989-1996	30-55y, F	326 CRA	326 polyp free controls	Incidence	NA	1,25(OH)$_2$D	Q4 vs Q1	OR 0.71(0.43-1.15)	1980-1990: BMI, physical activity, aspirin use, cigarette pack-years smoked, alcohol consumption, intake of red meat and methionine 1980: folic acid intake 1990: HRT.
								25(OH)D	Q4 vs Q1	OR 1.04(0.66-1.66)	
Almendingen, 2001, Norway	Case-control	3-year follow up	50-76y, M+F	87 CRA	35 healthy controls and 35 hospital controls	Recurrence	Total	NA	Highest vs lowest	Total	BMI, presence or absence of familial history of CRC among first-degree relatives, energy intake, fat intake, fiber intake and smoking status.
									Hospital control	OR 0.6(0.1-2.6)	
									Healthy control	OR 2.0(0.4-11.5)	
							Dietary		Highest vs lowest	Dietary	
									Hospital control	OR 0.2(0.1-0.9)	
									Healthy control	OR 0.4(0.1-2.2)	
Ingles, 2001,	Case-control	1991-1993	50-74y,	373 CRA	394 polyp-	Incidence	Dietary	NA	<270IU/d		BMI, total calorie intake total fat
									FF	OR	

USA			M+F		free controls					1.0	intake, total fiber intake, serum triglyceride level, calcium intake, vitamin D intake, NSAID usage, physical activity, smoking.
									Ff	OR 0.25(0.09-0.69)	
									ff	OR 0.22(0.04-1.20)	
									≧270IU/d		
									FF	OR 1.0	
									Ff	OR 1.50(0.66-3.60)	
									ff	OR 0.44(0.10-1.90)	
Kim, 2001, USA	Case-control	1991-1994	30-74y, M+F	393 CRA	406 polyp-free controls	Incidence	Total	NA	Lowest vs highest		Age, gender, HRT, total caloric intake, BMI, and smoking.
									Bb genotype	OR 1.40(0.73-2.70)	
Levine, 2001, USA	Case-control	1991-1993	50-74y, M+F	473 CRA	507 polyp-free controls	Incidence	Dietary	Plasma 25(OH)D	Highest vs lowest	Dietary Vitamin D	Age, gender, race, clinic, sigmoidoscopy date, calories, BMI, and total dietary fiber intake, saturated fat intake, and multivitamin use.
										OR 1.11(0.67-1.55)	
									Highest vs lowest	25(OH)D	
										OR 0.74(0.51-1.09)	

Martinez, 2002, USA	Cross-sectional	36.8± 16.0 months	40-80y, M+F	639 recurrence CRA	665 without recurrence	Recurrence	Dietary	NA	Highest vs lowest	Dietary	Age, gender, number of colonoscopies, history of polyps prior to baseline, aspirin use, dietary fiber, and total calcium intake.
										OR 0.78(0.54-1.13)	
							Supplementary		Highest vs lowest	Supplementary	
										OR 1.05(0.56-1.98)	
							Total		Highest vs lowest	Total	
										OR 1.02(0.71-1.47)	
Morimoto, 2002, USA	Case-control	1991-1994	30-74y, M+F	219 hyperplastic polyps, 437 adenomas, 138 both types of polyps	708 polyp-free controls	Incidence	Total	NA	Adenomatous polyps		Age, sex, BMI, percentage of kilocalories from fat, dietary fiber intake, HRT use, pack-years of smoking, dietary intake of folate, vitamin B6, vitamin B12, methionine, and alcohol.
									Highest vs lowest	OR 1.3(0.7-2.2)	
									Both type		
									Highest vs lowest	OR 0.5(0.2-1.2)	
Boyapati,	Case-control	1995-1997	30-74y,	177 CRA	228 polyp-	Incidence	Total	NA	High vs low	OR 0.69(0.41-1.18)	Age, sex, energy.

2003, USA			M+F		free controls						
Grau, 2003, USA	RCT	1988-1996	Mean age 61y, M+F	376 recurrent adenomas	803 CRA	Recurrence	Supplementary	25(OH)D	Calcium vs placebo		Age, sex, center, smoking status, and alcohol intake.
									≤29.1ng/ml	OR 1.05(0.85-1.29)	
									>29.1ng/ml	OR 0.71(0.57-0.89)	
								1,25(OH)D_2	Calcium vs placebo		
									≤41.8pg/ml	OR 0.84(0.69-1.03)	
									>41.8pg/ml	OR 0.91(0.72-1.14)	
Lieberman, 2003, USA	Cross-sectional	1994-1997	50-75y, M+F	329 advanced neoplasia	1770 participants	Incidence	Dietary	NA	Highest vs lowest	OR 0.61(0.39-0.97)	Age, total energy, medical history factors
Peters, 2004, USA	Case-control	1993-1999	55-74y, M+F	394 advanced adenomas	397 polyp-free controls	Incidence	NA	25(OH)D	Q5 vs Q1		Age, gender, ethnic origin, study center, and months of blood draw.
									Female	OR 0.27(0.11-0.69)	
									Male	OR 1.10(0.60-2.05)	
								1,25(OH)D_2	Q5 vs Q1		
									Female	OR 0.64(0.28-1.47)	

									Male	OR 1.13(0.62-2.04)	
Hartman, 2005, USA	RCT	1991-1994	≥35y, M+F	754 recurrent adenomas	1905 CRA	Recurrence	Dietary	NA	Q5 vs Q1	OR 0.93(0.69-1.25)	Age, NSAID use, gender, total energy intake, intervention assignment, location of clinical center, and gender-intervention group.
							Supplementary		Q5 vs Q1	OR 0.80(0.63-1.00)	
							Total		Q5 vs Q1	OR 0.84(0.62-1.13)	
Kesse, 2005, France	Cohort	EPIC 1993-1995	40-65y, F	516 CRA	16,489 participants	Incidence	Dietary	NA	Highest vs lowest	RR 1.15(0.88-1.49)	Age, educational level, current smoking status, family history of colon cancer, BMI, physical activity level and energy and alcohol intake.
Senesse, 2005, France	Case-control	NA	30-70y, M+F	362 CRA	427 polyp-free controls	NA	Dietary	NA	Q4 vs Q1	OR 0.6(0.4-1.0)	Age, sex, BMI, tobacco use, and energy and alcohol intakes.

Jacobs, 2007, USA	RCT	4-year follow-up	40-80y, M+F	Dietary vitamin D: 504 recurrent CRA; 25(OD)D: 211 recurrent CRA	Dietary vitamin D: 1192 participants of UDCA; Serum 25(OD) D: 568 participants of UDCA	Recurrence	Dietary	Serum 25(OH)D	Dietary Vitamin D		Gender, energy intake, number of colonoscopies, and previous polyps.
									Highest vs lowest	OR 1.00(0.68-1.47)	
									Serum 25(OH)D		BMI, number of colonoscopies, previous polyps, season of blood draw, and gender.
									Highest vs lowest	OR 0.74(0.46-1.17)	
Miller, 2006, USA	Cross-sectional	1998-2000	≥30y, M+F	222 CRA	479 adenoma-free controls	Incidence	NA	25(OH)D	Highest vs lowest	OR 0.51(0.27-0.98)	Age, sex, race, and month of Blood Draw.
Oh, 2007, USA	Cohort	NHS 1980-2002	30-55y, F	2747 CRA	48115 polyp-free controls	Incidence	Total	NA	Q5 vs Q1	RR 0.79(0.63-0.99)	Age, BMI, smoking, alcohol intake, family history of colon cancer, history of previous endoscopic screening, aspirin use, physical activity,

											menopausal status and hormone use, and energy, total fiber, red meat, folate, phosphorus, calcium, and retinol intake.
Hubner, 2008, USA	RCT	1997-2001	Mean age 57.5y, M+F	188 recurrent CRA	716 UKCAP participants	Recurrence	Dietary	NA	High vs low	RR 0.85(0.64-1.14)	Age, sex, aspirin and folate intervention, and interval between entry and follow-up colonoscopy.
							Total		High vs low	RR 0.96(0.72-1.27)	
Fedirko, 2010, USA	Case-control	1991-2002	Mean age 55.6y, M+F	616 CRA	770 polyp-free controls	Incidence	NA	Circulating 25(OH)D_3	Q4 vs Q1	OR 0.59(0.41-0.84)	Age, sex, family history of colorectal cancer in a first-degree relative, regular use of aspirin or NSAIDs, smoking, physical activity, body mass index, total red and processed meat intake,

											alcohol intake, calcium intake, retinol intake, and folate intake, study.
Ramadas, 2010, Malaysia	Case-control	2005-2006	≥30y, M+F	59 CRA	59 polyp-free controls	Incidence	Dietary	NA	T3 vs T1	OR 0.31(0.11-0.89)	Age, ethnicity, income, alcohol consumption, smoking status, energy intake.
Takahashi, 2010, Japan	Case-control	1997-2004	50y, M	656 CRA	648 polyp-free controls	Incidence	NA	Plasma or serum 25(OH)D	Q4 vs Q1	OR 1.25(0.85-1.84)	Hospital, rank in the Self Defense Forces, smoking, alcohol use, parental history of colorectal cancer, physical activity, BMI and type of blood sample, with stratification by month of blood drawing.
Adams, 2011, USA	Case-control	NA	<80y, M+F	63 CRA	180 polyp-free controls	Incidence	NA	Plasma total 25(OH)D	Highest vs lowest	OR 0.52(0.23-1.20)	Age, sex, previous polyp diagnosis, season of blood draw, BMI,

											history of cigarette use, supplement use, and physical activity level.
LePane, 2011, USA	Cross-sectional	2009	≥18y, M+F	124 CRA	527 polyp-free controls	Incidence	NA	Serum 25(OH)D	Highest vs lowest		
Hong, 2012, South Korea	Case-control	2009-2010	50-74y, M+F	143 CRA	143 polyp-free controls	Incidence	NA	Serum 25(OH)D3	Q4 vs Q1	OR 0.38(0.18-0.80)	Age, sex, BMI, smoking, alcohol drinking, physical activity and corrected calcium level.
Yamaji, 2012, Japan	Case-control	2004-2005	50-79y, M, 40-79y, F	737 CRA	703 benign lesions	Incidence	NA	Plasma 25(OH)D	Q5 vs Q1	OR 0.64(0.45-0.92)	Sex, age, screening period, and season of blood collection, cigarette smoking, alcohol drinking, BMI, family history of colorectal cancer, NSAID use, daily energy intake, and

											height.
Heine, 2013, Netherland	Cohort	1995-2008	18-75y, M+F	165 recurrent CRA	565 CRA	Recurrence	Supplementary	NA	User vs nonuser	HR 0.83(0.45-1.50)	Age, sex, educational level, number of colonoscopies during follow-up.
Jacobs, 2013, USA	RCT	WBF: 1990-1995 UDCA: 1995-1999	Median 67.0y, M+F	942 with recurrent CRA	2074 CRA	Incidence	NA	25(OH)D	Incidence	Large size	BMI, gender, age, race and study (for pooled analysis only).
									Highest vs lowest	OR 0.83(0.64-1.08)	
						Recurrence			Recurrence		
									Highest vs lowest	OR 0.95(0.73-1.24)	
Aigner, 2014, Australia	Cross-sectional	2010-2013	33-87y, M, 31-88y, F	436 CRA	1538 participants	Incidence	Supplementary	Serum 25(OH)D3	Highest vs lowest		
Hibler, 2014, USA	RCT	At least 6 months follow-up	40-80y, M+F	485 metachronous adenomas	1151 UDCA participants	Recurrence	NA	$1,25(OH)_2D$	T3 vs T1	OR 0.81(0.60-1.10)	BMI, gender, and age.
Yang, 2014,	Case-control	1991-2002	30-74y,	401 CRA	518 polyp-	Incidence	NA	25(OH)D3	T3 vs T1	OR 0.77(0.52-1.13)	Age, sex, family history of

USA			M+F		free controls						colorectal cancer in a 1st degree relative, ever-smoking, and NSAID use.
Baron, 2015, USA	RCT	2004-2008	45-75y, M+F	880 recurrent CRA	2259 CRA	Recurrence	Supplementary	Serum 25(OH)D	Supplementary Vitamin D		Age, clinical center, anticipated surveillance interval, a three-level variable for sex and type of randomization, and number of baseline adenomas.
									User vs nonuser	RR 0.99(0.89-1.09)	
									25(OH)D		Age, clinical center, anticipated surveillance interval, a three-level variable for sex and type of randomization, number of baseline adenomas, and calcium treatment
									Q4 vs Q1	RR 0.98(0.79-1.21)	

											assignment.
Hibler, 2015, USA	RCT	At least 6 months follow-up	Mean age 65.6y, M+F	519 recurrent CRA	1188 CRA	Recurrence	NA	25(OH)D	Highest vs lowest		Age and sex.
									AA	OR 0.78(0.27-2.30)	
									AG	OR 0.21(0.07-0.62)	
									GG	OR 0.18(0.03-0.91)	
								$1,25(OH)_2D$	Highest vs lowest		
									GG	OR 0.98(0.63-1.53)	
									GC	OR 0.54(0.30-0.95)	
									CC	OR 0.49(0.10-2.46)	
Ahmad, 2016, USA	Case-control	NA	Mean age 61.2y, M+F	40 advanced adenomas	139 polyp-free controls	Incidence	NA	25(OH)D	Lowest vs highest	OR 2.1(0.6-7.3)	Age.
Jacobs, 2016, USA	Cross-sectional	NA	40-80y, M+F	485 recurrent CRA	1150 CRA	Recurrence	NA	25(OH)D	Highest vs lowest		
								$1,25(OH)_2D$			
Barry, 2017,	RCT	2004-2013	40-75y,	79 recurrent	835 CRA	Recurrence	NA	25(OH)D	Q4 vs Q1		

USA			M+F	advanced adenomas							
Bryce, 2018, USA	Cohort	2014-2015	≥18y, M+F	104 CRA	228 participants	Incidence	NA	25(OH)D	Lowest vs highest	OR 1.05(0.48-2.32)	Season of blood draw, race, sex, BMI, age, current smoking, and any current vitamin D or calcium use.
Gibbs, 2018, USA	Case-control	CRPU: 1991-1994, MAPI:1994-1997, MAPII: 2002	Mean age 54.2y, M+F	418 CRA	524 polyp-free controls	Incidence	NA	25(OH)D	Highest vs lowest		Age, sex, study, regular use of aspirin or NSAIDs, family history of colorectal cancer in a first-degree relative, smoking status, alcohol intake, total calcium intake from diet and supplements, BMI, and physical activity.
									Gc1-1	OR 1.02(0.55-1.91)	
									Gc1-2/Gc2-2	OR 0.46(0.24-0.88)	

He, 2018, USA	Cohort	18-20 years follow-up	NHS: 30-55y, F, NHS2: 25-42y, F, HPFS: 40-75y, M	9212 with conventional adenoma, 2382 with synchronous SPs and conventional adenomas	53858 NHS participants, 58574 NHS2 participants, 28711 HPFS participants	Incidence	Total	NA	Q4 vs Q1		Cohort, time period of endoscopy, number of prior endoscopies, time in years since the most recent endoscopy, reason for endoscopy, race, age, family history of colorectal cancer, pack-years of smoking, BMI, physical activity, alcohol intake, height, regular aspirin use. For dietary factors, test for trend was conducted using the median of each quartile as a continuous variable.
									Conventional adenoma only	OR 0.85(0.80-0.90)	
									SPs and conventional adenomas	OR 0.85(0.76-0.96)	
Calder	RCT	3-5	45-	569	1121	Recurrence	Supplementary	25(OH)D	User vs	RR	Age, clinical

wood, 2019, Germany		years follow-up	75y, M+F	recurrent CRA	CRA				nonuser	1.04(0.93-1.17)	center, anticipated surveillance interval, three-level variable for sex and randomization arm, number of baseline adenomas.
Crockett, 2019, USA	RCT	3-5 years follow-up	45-75y, M+F	138 SSA/P	2259 SPs	Recurrence	Supplementary	25(OH)D	Supplementary Vitamin D		Age, sex, clinical center, race, BMI, smoking status, anticipated surveillance interval, randomization arm of randomization, number of baseline serrated polyps.
									User vs nonuser	RR 1.31(0.82-2.10)	
									25(OH)D		
Chatterjee, 2021, USA	RCT	Median follow-up 2.9	Mean age 60y, M+F	239 CRA	2385 participants	Incidence	Supplementary	NA	User vs nonuser	HR 0.83(0.64-1.07)	Site, BMI and race.

		years									
Hellwege, 2021, USA	Nested case-control	1993-2001	55-74y, M+F	755 incident CRA and 755 metachronous adenoma	755 polyp-free controls and 755 CRA	Incidence	NA	25(OH)D	Incidence		Age, sex, race, BMI, education, family history of colorectal cancer, cigarette, alcohol drinking status, hours spent in vigorous activities, intake of total energy, calcium, magnesium, vitamin D, vitamin C, beta-carotene, saturated fatty acid, processed meat, season of blood draw, time of day of blood draw, latitude region, use of aspirin, and use of ibuprofen.
									Lowest vs highest	1.10(0.64-1.89)	
						Metachronous			Metachronous		
									Lowest vs highest	1.06(0.61-1.84)	
Kim,	Cohort	1991-	<50y,	1439	29,186	Incidence	Total		Highest vs	OR	Age, time period

2021, USA		2011	F	conventional adenoma	participants				lowest	0.71(0.56-0.89)	of endoscopy, time since most recent endoscopy, number of reported endoscopies, reason for current endoscopy, race, height, BMI, alcohol intake, smoking, regular use of aspirin, regular use of NSAIDs, physical activity, TV viewing time, family history of colorectal cancer, and dietary intake.
Song, 2020, USA	RCT	2011-2017	≥50y, M, ≥55y, F	308 CRA in vitamin group, 287 CRA in placebo	12,927 participants in vitamin group,	Incidence	Supplementary	NA	Incidence		Age, sex, fish oil treatment assignment, and use of colonoscopy or
									User vs nonuser	OR 1.10(0.93-1.30)	
						Recurrence			Recurrence		

		group	12,944 participants in placebo group			User vs nonuser	OR 0.75(0.35-1.58)	sigmoidoscopy in the past 10 years before randomization.

NHS: Nurse Health Study; HPFS: Health Professionals Follow-up Study; EPIC: European Prospective Investigation into Cancer and Nutrition; UKCAP: United Kingdom Colorectal Adenoma Prevention; WBF: Wheat Bran Fiber Trial; UDCA: Ursodeoxycholic Acid Trial; CRPU: Cancer Prevention Research Unit; MAPI: Markers of Adenomatous Polyps-I Study; MAPII: Markers of Adenomatous Polyps-II Study; VITAL: Vitamin D and Omega-3 Trail; SP: serrated polyp; SSA/P: sessile serrated adenomas or polyp; BMI: body mass Index; NSAID: nonsteroidal anti-inflammatory drug; HRT: Hormone replacement therapy; NA: Not applicable.

Supplementary Figure 1. Risk of bias summary and graph.

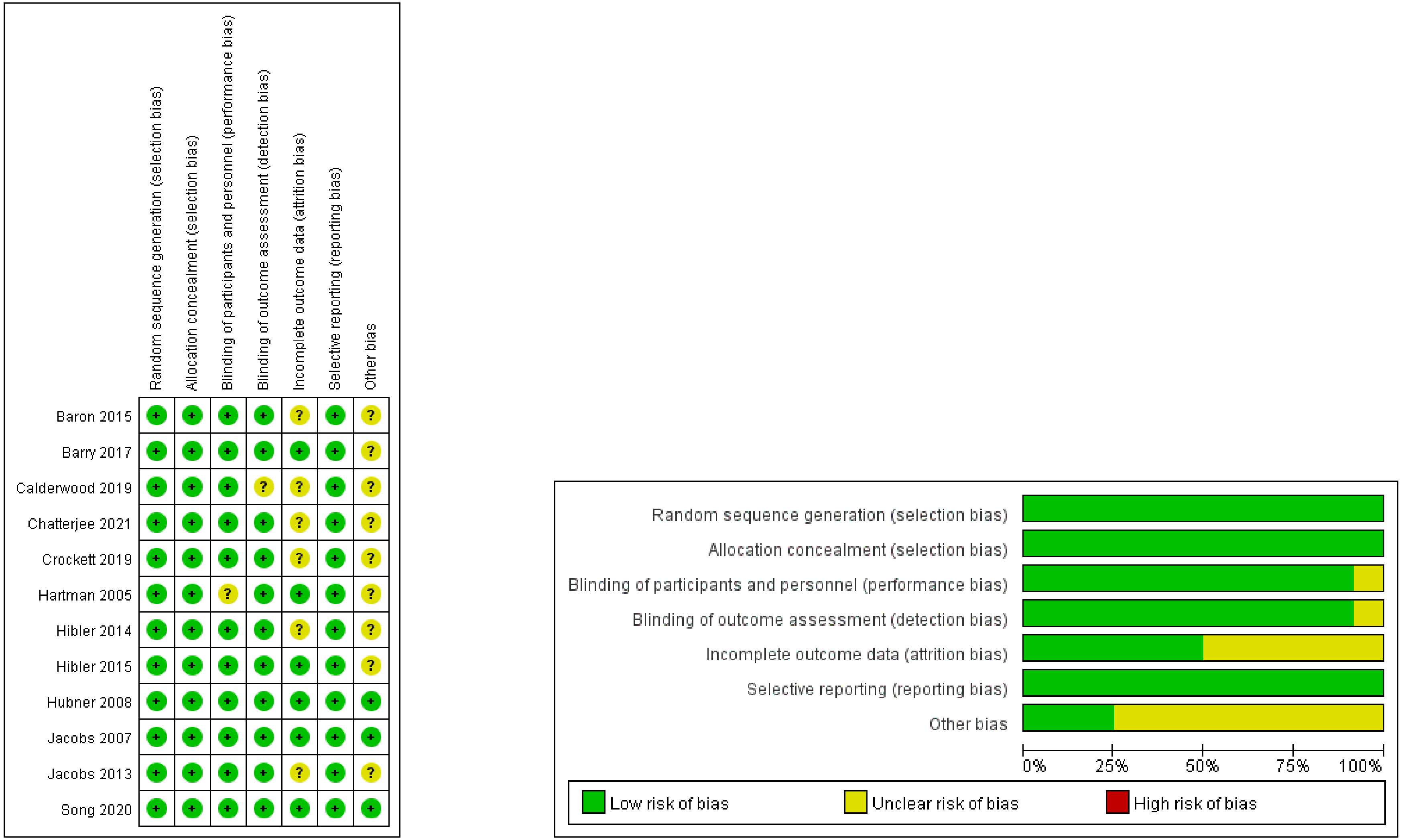

Supplementary Figure 2. Forrest plot of subgroup analyses. (a) Total vitamin D intake and risk of CRC precursors incidence stratified by lesion type; (b) Total vitamin D intake and risk of CRC precursors incidence stratified by lesion location; (c) Circulating 25(OH)D level and risk of CRC precursors incidence stratified by sex; (d) Circulating 25(OH)D level and risk of CRC precursors incidence stratified by lesion type; (e) Circulating 25(OH)D level and risk of CRC precursors incidence stratified by lesion location.

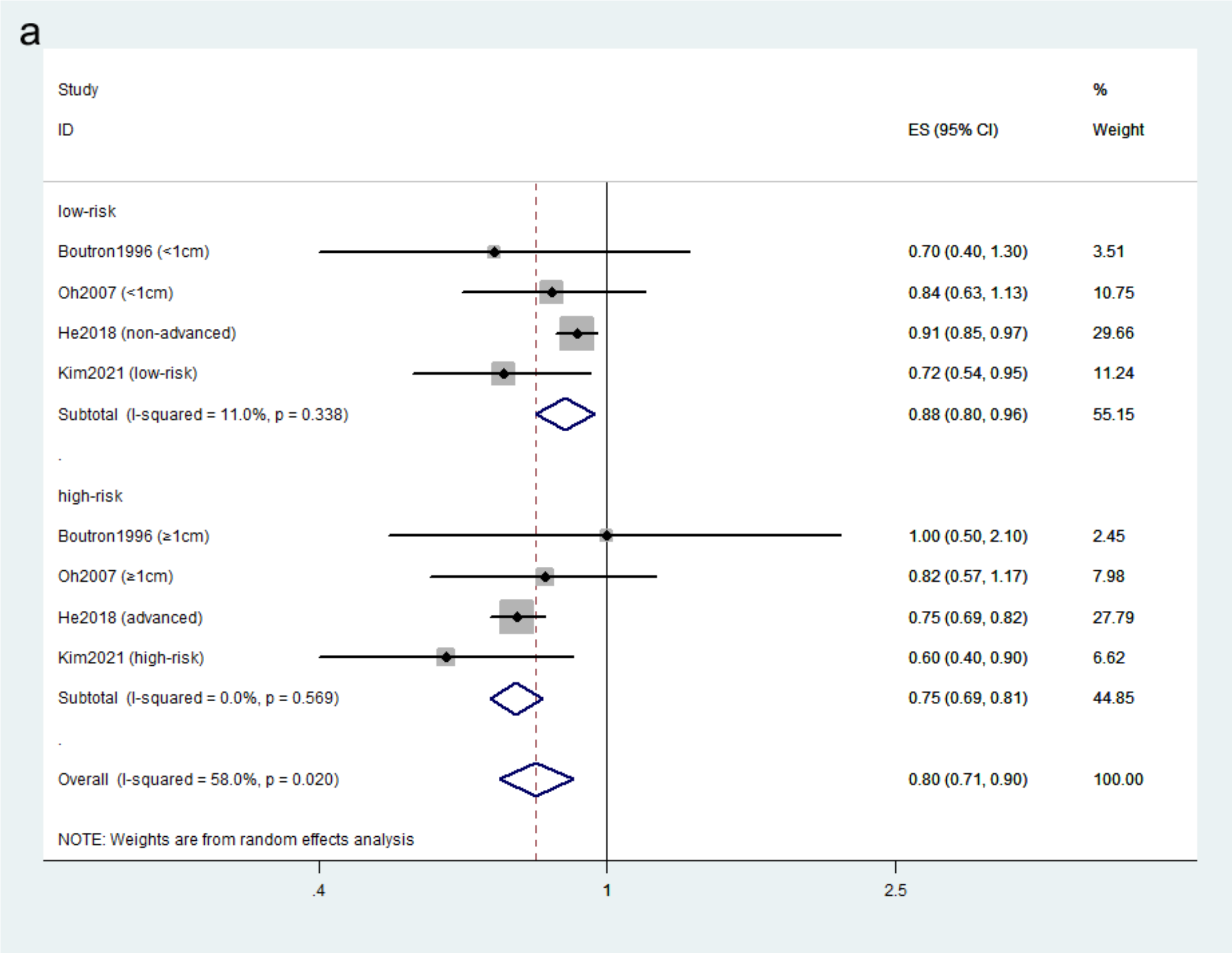

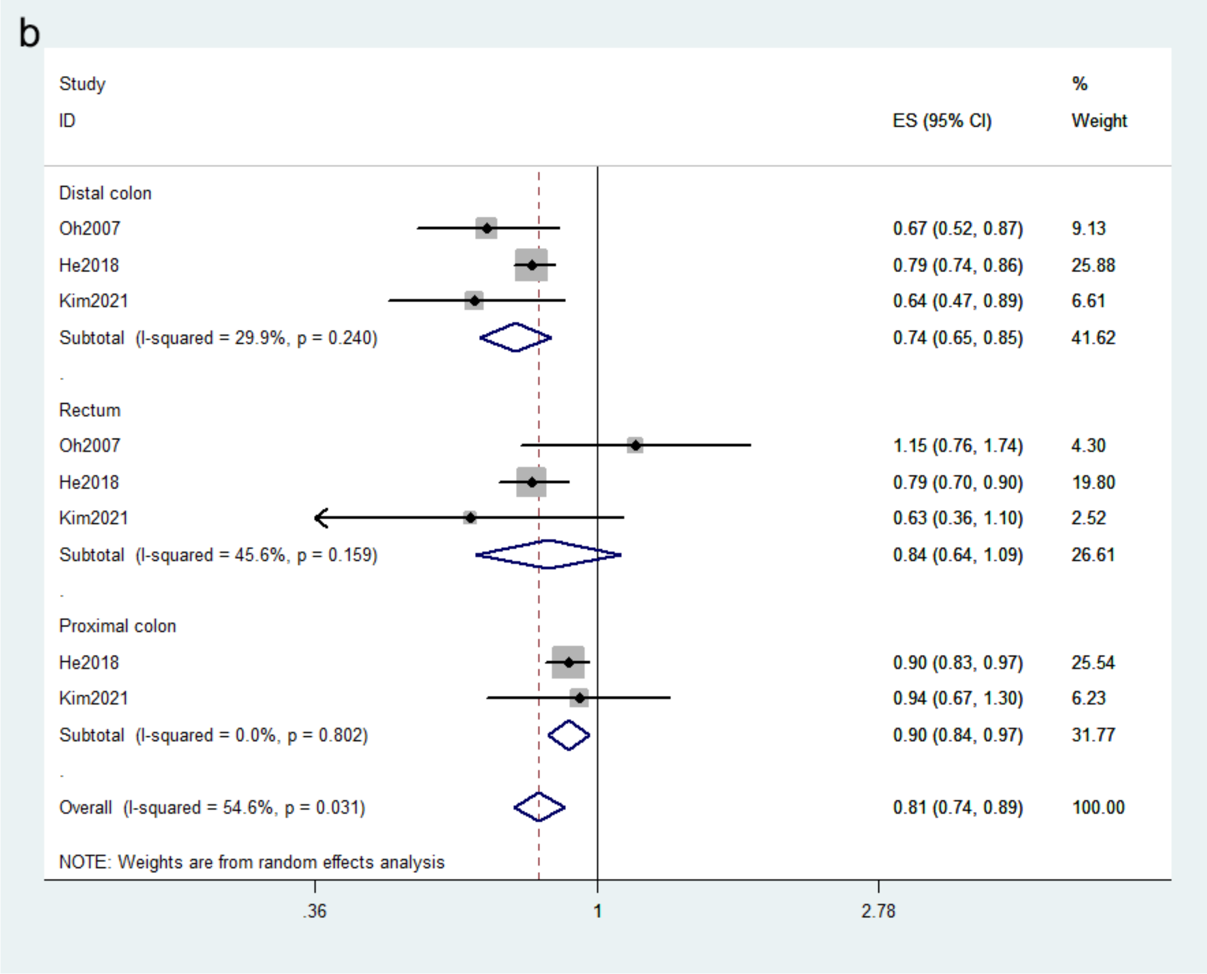
b
Study
ID
ES (95% CI)
%
Weight
Distal colon
Oh2007
0.67 (0.52, 0.87)
9.13
He2018
0.79 (0.74, 0.86)
25.88
Kim2021
0.64 (0.47, 0.89)
6.61
Subtotal (I-squared = 29.9%, p = 0.240)
0.74 (0.65, 0.85)
41.62
Rectum
Oh2007
1.15 (0.76, 1.74)
4.30
He2018
0.79 (0.70, 0.90)
19.80
Kim2021
0.63 (0.36, 1.10)
2.52
Subtotal (I-squared = 45.6%, p = 0.159)
0.84 (0.64, 1.09)
26.61
Proximal colon
He2018
0.90 (0.83, 0.97)
25.54
Kim2021
0.94 (0.67, 1.30)
6.23
Subtotal (I-squared = 0.0%, p = 0.802)
0.90 (0.84, 0.97)
31.77
Overall (I-squared = 54.6%, p = 0.031)
0.81 (0.74, 0.89)
100.00
NOTE: Weights are from random effects analysis
.36
1
2.78

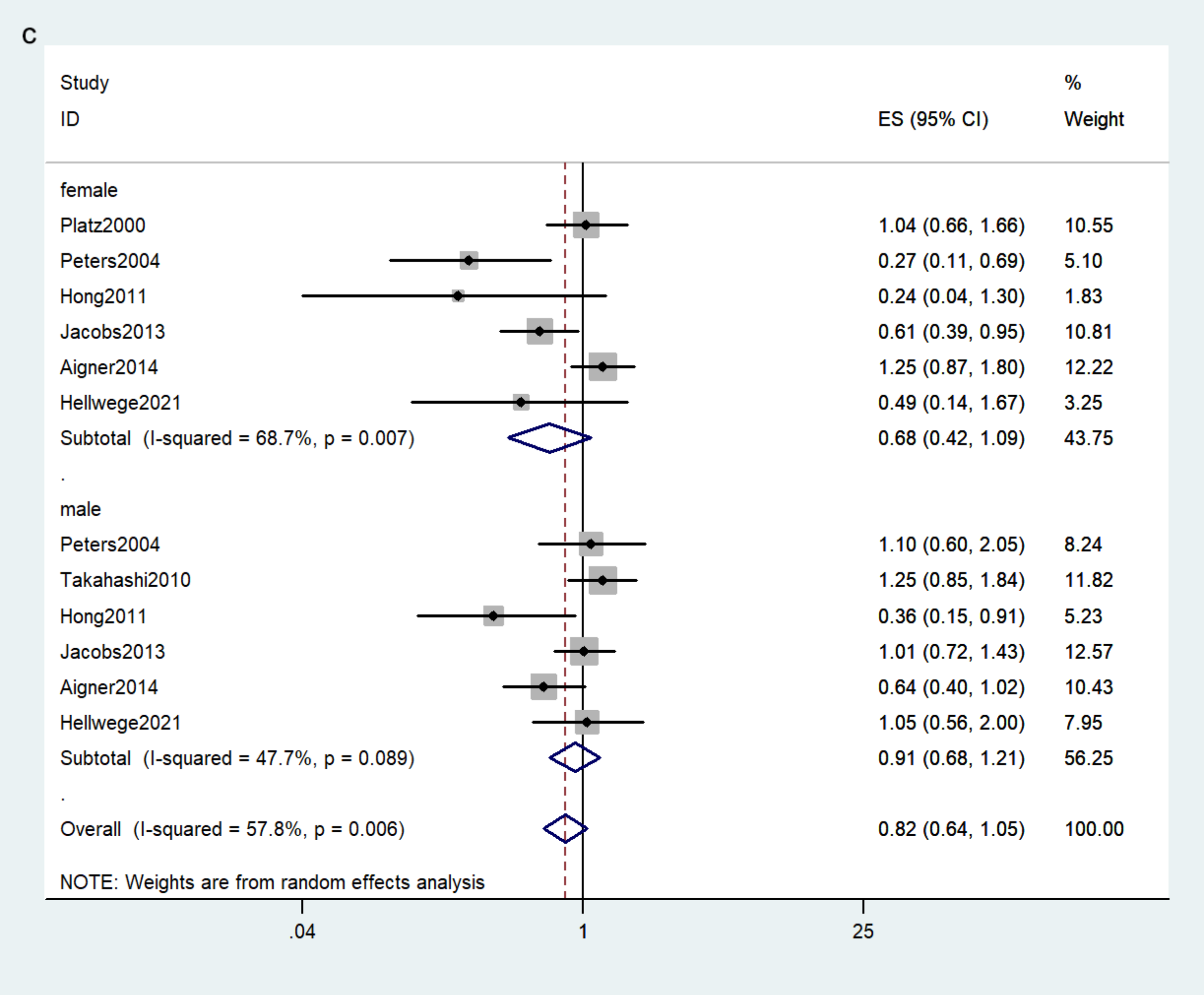
C
Study
ID
ES (95% CI)
%
Weight
female
Platz2000 1.04 (0.66, 1.66) 10.55
Peters2004 0.27 (0.11, 0.69) 5.10
Hong2011 0.24 (0.04, 1.30) 1.83
Jacobs2013 0.61 (0.39, 0.95) 10.81
Aigner2014 1.25 (0.87, 1.80) 12.22
Hellwege2021 0.49 (0.14, 1.67) 3.25
Subtotal (I-squared = 68.7%, p = 0.007) 0.68 (0.42, 1.09) 43.75
male
Peters2004 1.10 (0.60, 2.05) 8.24
Takahashi2010 1.25 (0.85, 1.84) 11.82
Hong2011 0.36 (0.15, 0.91) 5.23
Jacobs2013 1.01 (0.72, 1.43) 12.57
Aigner2014 0.64 (0.40, 1.02) 10.43
Hellwege2021 1.05 (0.56, 2.00) 7.95
Subtotal (I-squared = 47.7%, p = 0.089) 0.91 (0.68, 1.21) 56.25
Overall (I-squared = 57.8%, p = 0.006) 0.82 (0.64, 1.05) 100.00
NOTE: Weights are from random effects analysis
.04
1
25

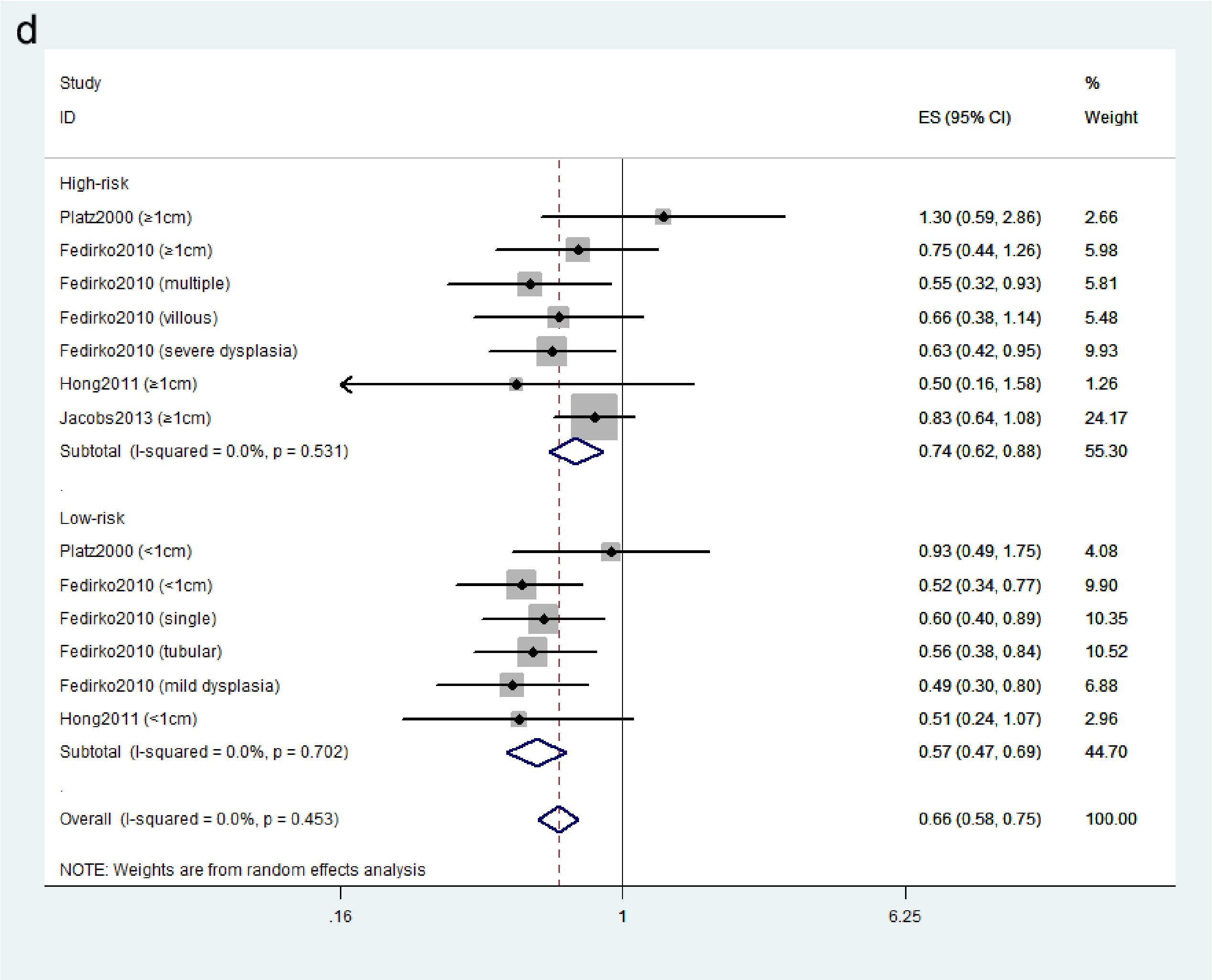

d
Study
ID
ES (95% CI)
%
Weight
High-risk
Platz2000 (≥1cm)
1.30 (0.59, 2.86)
2.66
Fedirko2010 (≥1cm)
0.75 (0.44, 1.26)
5.98
Fedirko2010 (multiple)
0.55 (0.32, 0.93)
5.81
Fedirko2010 (villous)
0.66 (0.38, 1.14)
5.48
Fedirko2010 (severe dysplasia)
0.63 (0.42, 0.95)
9.93
Hong2011 (≥1cm)
0.50 (0.16, 1.58)
1.26
Jacobs2013 (≥1cm)
0.83 (0.64, 1.08)
24.17
Subtotal (I-squared = 0.0%, p = 0.531)
0.74 (0.62, 0.88)
55.30
Low-risk
Platz2000 (<1cm)
0.93 (0.49, 1.75)
4.08
Fedirko2010 (<1cm)
0.52 (0.34, 0.77)
9.90
Fedirko2010 (single)
0.60 (0.40, 0.89)
10.35
Fedirko2010 (tubular)
0.56 (0.38, 0.84)
10.52
Fedirko2010 (mild dysplasia)
0.49 (0.30, 0.80)
6.88
Hong2011 (<1cm)
0.51 (0.24, 1.07)
2.96
Subtotal (I-squared = 0.0%, p = 0.702)
0.57 (0.47, 0.69)
44.70
Overall (I-squared = 0.0%, p = 0.453)
0.66 (0.58, 0.75)
100.00
NOTE: Weights are from random effects analysis
.16
1
6.25

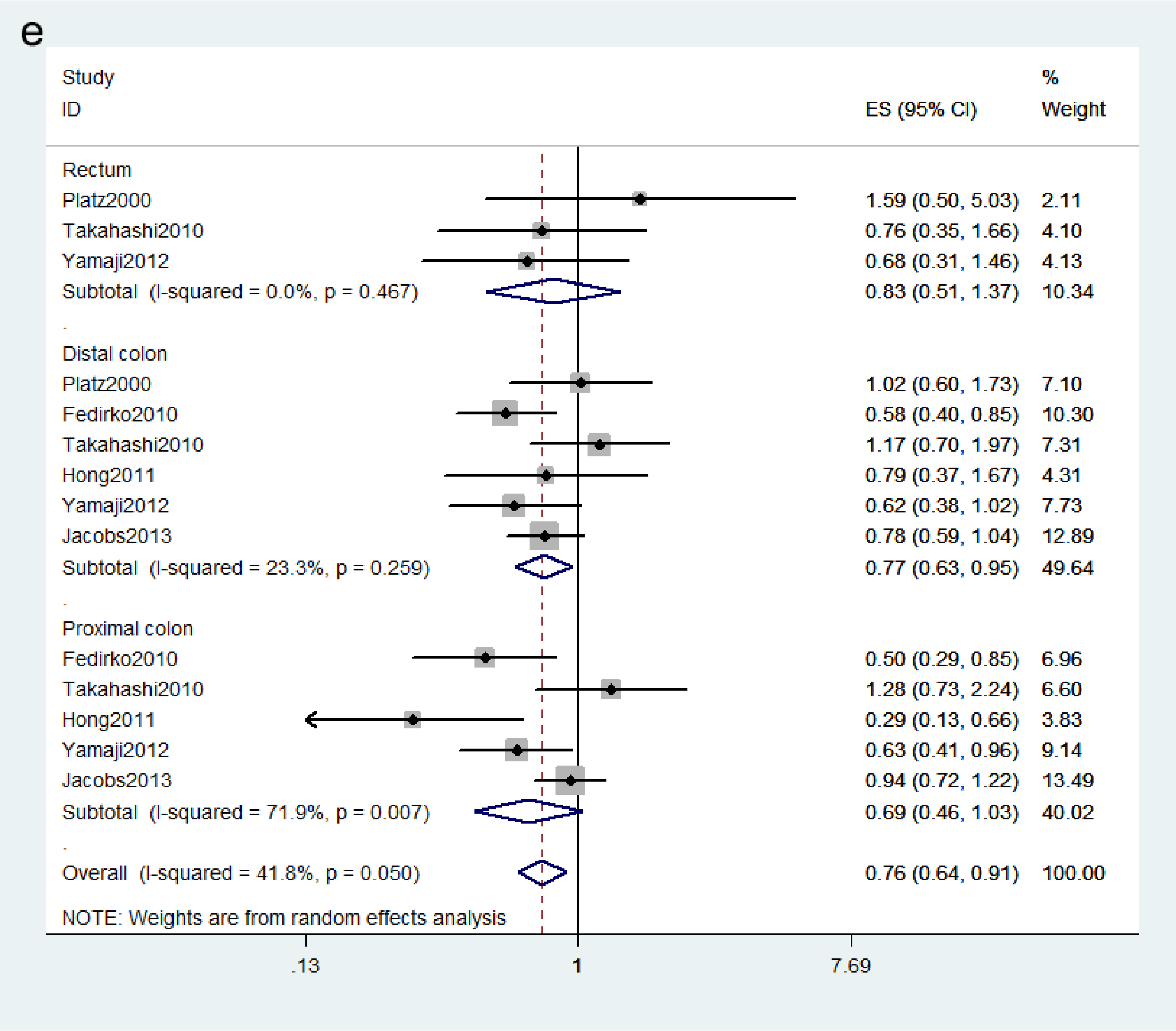
e
Study
ID
ES (95% CI)
%
Weight
Rectum
Platz2000
1.59 (0.50, 5.03)
2.11
Takahashi2010
0.76 (0.35, 1.66)
4.10
Yamaji2012
0.68 (0.31, 1.46)
4.13
Subtotal (I-squared = 0.0%, p = 0.467)
0.83 (0.51, 1.37)
10.34
Distal colon
Platz2000
1.02 (0.60, 1.73)
7.10
Fedirko2010
0.58 (0.40, 0.85)
10.30
Takahashi2010
1.17 (0.70, 1.97)
7.31
Hong2011
0.79 (0.37, 1.67)
4.31
Yamaji2012
0.62 (0.38, 1.02)
7.73
Jacobs2013
0.78 (0.59, 1.04)
12.89
Subtotal (I-squared = 23.3%, p = 0.259)
0.77 (0.63, 0.95)
49.64
Proximal colon
Fedirko2010
0.50 (0.29, 0.85)
6.96
Takahashi2010
1.28 (0.73, 2.24)
6.60
Hong2011
0.29 (0.13, 0.66)
3.83
Yamaji2012
0.63 (0.41, 0.96)
9.14
Jacobs2013
0.94 (0.72, 1.22)
13.49
Subtotal (I-squared = 71.9%, p = 0.007)
0.69 (0.46, 1.03)
40.02
Overall (I-squared = 41.8%, p = 0.050)
0.76 (0.64, 0.91)
100.00
NOTE: Weights are from random effects analysis
.13
1
7.69

Supplementary Figure 3. Funnel plot for publication bias for vitamin D intake, circulating 25(OH)D level and incidence and recurrence of CRC precursors. (a) Total vitamin D intake and incidence of CRC precursors; (b) Dietary vitamin D and incidence of CRC precursors; (c) Supplementary vitamin D intake and recurrence of CRC precursors; (d) Circulating 25(OH)D level and incidence of CRC precursors; (e) Circulating 25(OH)D level and incidence of CRC precursors in case-control studies; (f) Circulating 25(OH)D level and incidence of CRC precursors in American populations; (g) Circulating 25(OH)D level and recurrence of CRC precursors; (h) Circulating 25(OH)D level and recurrence of CRC precursors in RCT studies; (i) Circulating 25(OH)D level and recurrence of CRC precursors in American populations.

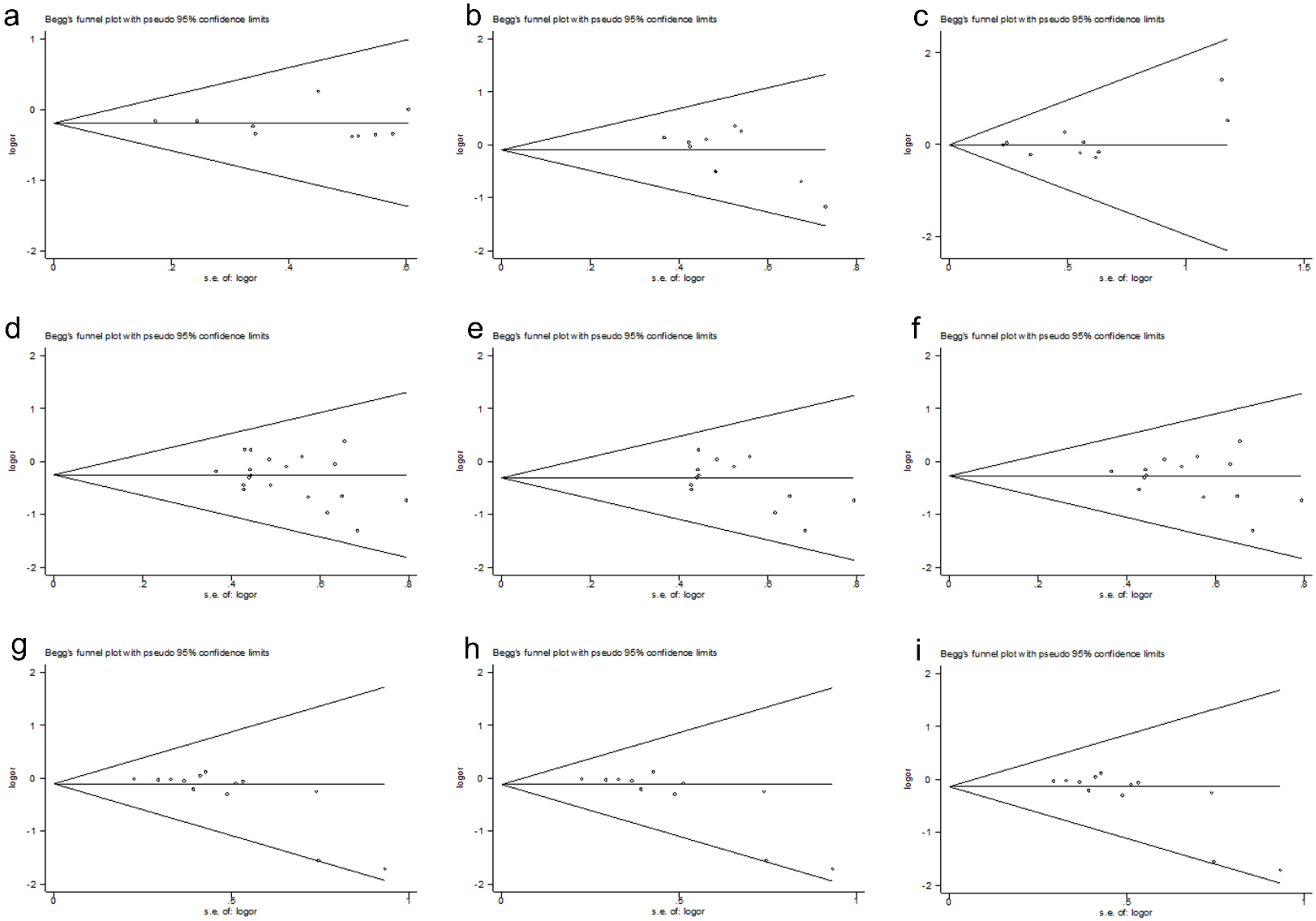
a
Begg's funnel plot with pseudo 95% confidence limits
logor
s.e. of: logor
b
Begg's funnel plot with pseudo 95% confidence limits
logor
s.e. of: logor
c
Begg's funnel plot with pseudo 95% confidence limits
logor
s.e. of: logor
d
Begg's funnel plot with pseudo 95% confidence limits
logor
s.e. of: logor
e
Begg's funnel plot with pseudo 95% confidence limits
logor
s.e. of: logor
f
Begg's funnel plot with pseudo 95% confidence limits
logor
s.e. of: logor
g
Begg's funnel plot with pseudo 95% confidence limits
logor
s.e. of: logor
h
Begg's funnel plot with pseudo 95% confidence limits
logor
s.e. of: logor
i
Begg's funnel plot with pseudo 95% confidence limits
logor
s.e. of: logor
